# Production of selenium nanoparticles occurs through an interconnected pathway of sulphur metabolism and oxidative stress response in *Pseudomonas putida* KT2440

**DOI:** 10.1111/1751-7915.14215

**Published:** 2023-01-22

**Authors:** Roberto Avendaño, Said Muñoz‐Montero, Diego Rojas‐Gätjens, Paola Fuentes‐Schweizer, Sofía Vieto, Rafael Montenegro, Manuel Salvador, Rufus Frew, Juhyun Kim, Max Chavarría, Jose I. Jiménez

**Affiliations:** ^1^ Centro Nacional de Innovaciones Biotecnológicas (CENIBiot), CeNAT‐CONARE San José Costa Rica; ^2^ Department of Life Sciences Imperial College London London UK; ^3^ Escuela de Química Universidad de Costa Rica San José Costa Rica; ^4^ Centro de Electroquímica y Energía Química (CELEQ) Universidad de Costa Rica San José Costa Rica; ^5^ Biotechnology Applications, IDENER Research & Development Seville Spain; ^6^ Department of Chemistry University of Leicester Leicester UK; ^7^ School of Life Sciences, BK21 FOUR KNU Creative BioResearch Group KNU Institute for Microorganisms, Kyungpook National University Daegu Korea; ^8^ Centro de Investigaciones en Productos Naturales (CIPRONA) Universidad de Costa Rica San José Costa Rica

## Abstract

The soil bacterium *Pseudomonas putida* KT2440 has been shown to produce selenium nanoparticles aerobically from selenite; however, the molecular actors involved in this process are unknown. Here, through a combination of genetic and analytical techniques, we report the first insights into selenite metabolism in this bacterium. Our results suggest that the reduction of selenite occurs through an interconnected metabolic network involving central metabolic reactions, sulphur metabolism, and the response to oxidative stress. Genes such as *sucA*, D2HGDH and PP_3148 revealed that the 2‐ketoglutarate and glutamate metabolism is important to convert selenite into selenium. On the other hand, mutations affecting the activity of the sulphite reductase decreased the bacteria's ability to transform selenite. Other genes related to sulphur metabolism (*ssuEF*, *sfnCE*, *sqrR*, *sqr* and *pdo2*) and stress response (*gqr*, *lsfA*, *ahpCF* and *sadI*) were also identified as involved in selenite transformation. Interestingly, suppression of genes *sqrR*, *sqr* and *pdo2* resulted in the production of selenium nanoparticles at a higher rate than the wild‐type strain, which is of biotechnological interest. The data provided in this study brings us closer to understanding the metabolism of selenium in bacteria and offers new targets for the development of biotechnological tools for the production of selenium nanoparticles.

## Graphical abstract

The soil bacterium *Pseudomonas putida* KT2440 is able to reduce selenite to form nanoparticles of elemental selenium. In this work we combine a transposition screening with transcriptomics and biochemical assays to shed light into this process of biotechnological interest. We report that selenite reduction depends on the intracellular availability of reduced glutathione and involves the concerted action of enzymes belonging to the sulphur metabolism, the central metabolism and oxidative stress pathways.

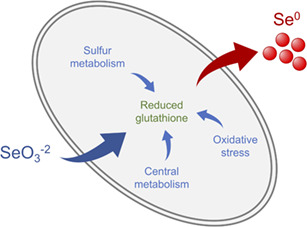

## INTRODUCTION

Selenium (Se) is a metalloid element with multiple applications in health and industry. Nanometric selenium has shown varied biological activities, including anticancer, antibacterial and protective agent for DNA against UV damage (Cremonini et al., 2016; Khurana et al., 2019; Rayman, 2008). In industry, elemental selenium is used to manufacture fertilizers, semiconductors and sensors or to be combined with other metals for electronics, imaging or photovoltaics (Chaudhary & Mehta, 2014). Considering the increased interest in using selenium in high‐tech applications (Nayak et al., 2021), treatment systems should not only aim to remove dissolved selenium but also consider recovering valuable nanoparticles in order to transition to a circular economy. In this sense, one of the methodologies that have recently attracted attention is the use of microorganisms to carry out the bioreduction of selenium oxyanions (i.e. selenate, selenite) to elemental selenium.

Several environmental bacteria have shown the ability to perform selenate and selenite reduction (Staicu & Barton, 2021). A representative example is *Thauera selenatis* (Macy et al., 1993), which reduces selenate to selenite via anaerobic respiration. Other bacteria from different genera have demonstrated the ability to reduce selenite to elemental selenium aerobically. These include species of *Pseudomonas* (Avendaño et al., 2016), *Comamonas* (Tan et al., 2018; Zheng et al., 2014), *Enterobacter* (Song et al., 2017), *Azospirillum* (Tugarova et al., 2020), *Bacillus* (Lampis et al., 2014) *Shewanella* (Klonowska et al., 2005; Li et al., 2014) and *Burkholderia* (Khoei et al., 2017) among others.

Despite many reports have shown the potential of bacteria for the treatment of water and soil contaminated with selenium, many aspects of how bacteria carry out this bioreduction process are still unclear. In most cases, only a few pieces of the metabolic puzzle are known and studies have been limited to bacteria such as *Escherichia coli*, *Salmonella typhimurium*, *Rhodobacter sphaeroides* and *Thauera selenatis* among others. Published works point to different selenate and selenite reduction mechanisms that include oxidative stress response (Bébien et al., 2001, 2002; Kessi, 2006; Tan et al., 2018; Yasir et al., 2020), sulphur metabolism (Tan et al., 2018; Yasir et al., 2020), as well as the participation of selenate and nitrite reductases in anaerobic bacteria (Butler et al., 2012; Debieux et al., 2011; DeMoll‐Decker & Macy, 1993; Krafft et al., 2000; Schröder et al., 1997). In *E. coli* and *Rhodospirillum rubrum* it has been shown that glutathione is a fundamental actor in a process combining abiotic chemical reactions and enzymes (Kessi & Hanselmann, 2004).

**FIGURE 1:**
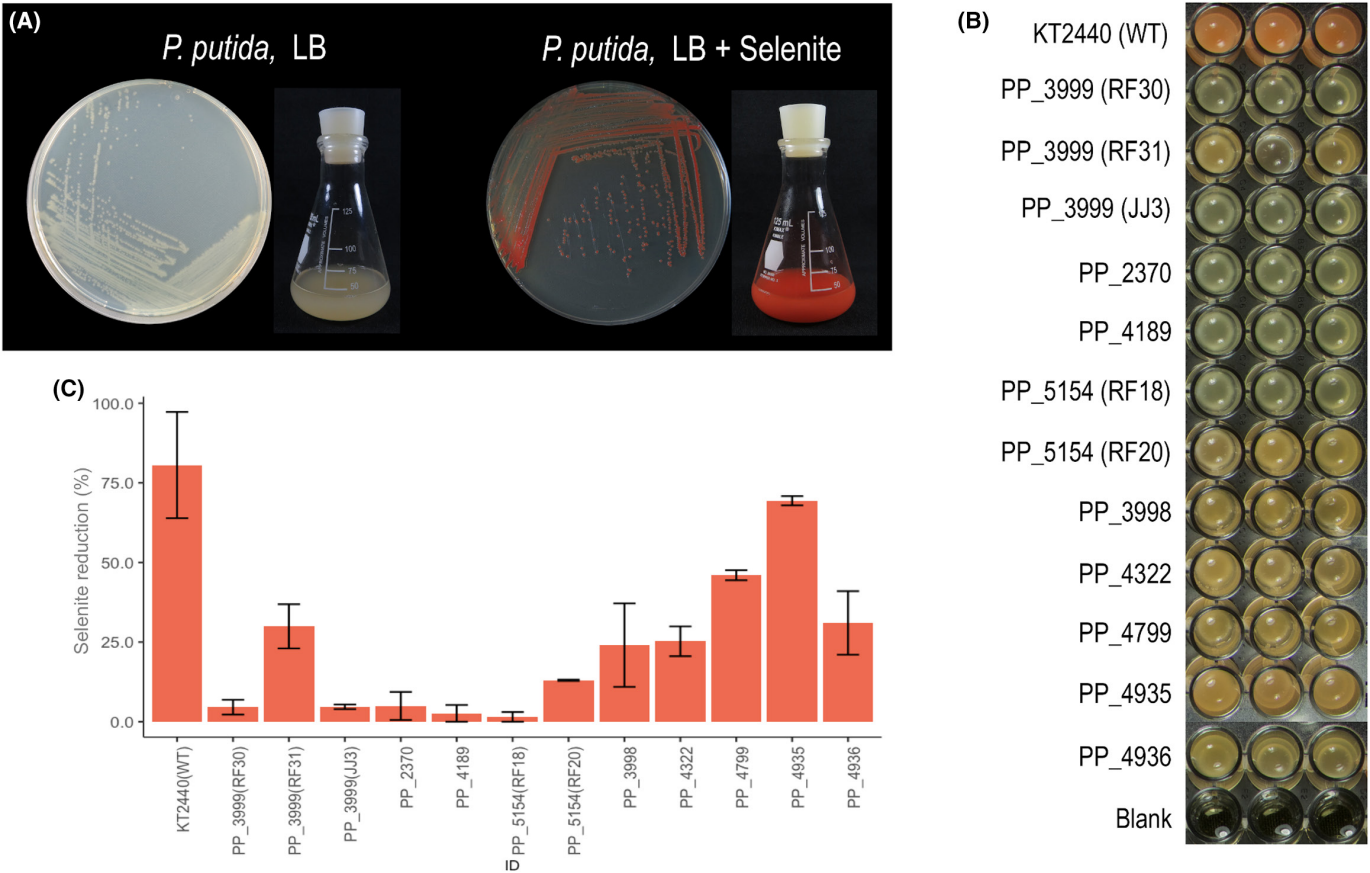
Growth of *Pseudomonas putida* KT2440 and Se‐delayed mutants in lysogeny broth (LB) broth in the presence of selenite (1 mM). (A) Photographs of *P. putida* KT2440 (WT) in both solid and liquid LB medium in the absence and presence of selenite. The photographs were taken after 24 h of growth. (B) Photographs of *P. putida* KT2440 (WT) and the 12 Se‐delayed mutants grown in LB culture medium in a microplate after 24 h. (C) Percentages of conversion of selenite to selenium by *P. putida* KT2440 and the 12 Se‐delayed mutants in LB culture medium after 24 h of growth. The average of three replicates is presented as a bar chart and the error was calculated as the standard deviation of the results. Percentages were calculated against the maximum reduction determined for the WT strain.

In general terms, there is a partial knowledge of mechanisms of reduction of selenium oxyanions in bacteria; however, there are many other cases of species with potential and very attractive characteristics for the bioremediation of selenium, for which there is no mechanistic information available. One of them is *Pseudomonas putida* KT2440, a bacterium whose ability to reduce selenite to selenium results in a distinct red phenotype (exemplified by Figure 1A; Avendaño et al., 2016). This soil bacterium is particularly interesting in biotechnology and specifically in reducing processes (Nikel & de Lorenzo, 2018; Nikel et al., 2016). One of these characteristics is that its central carbon metabolism is adapted to produce a high reducing power (Chavarría et al., 2013; Nikel et al., 2015, 2021), due to a sugar catabolism funnelled through the EDEMP cycle that directs the carbon flux to NADPH‐forming reactions (Nikel & Chavarría, 2015). The resulting NADPH is responsible for maintaining glutathione in its reduced form, and as a consequence, *P. putida* has high reducing power and greater resistance to oxidative stress (Chavarría et al., 2013; Nikel et al., 2015, 2021). This metabolic capacity could be used for the implementation of reduction reactions such as the transformation of oxyanions (e.g. selenite, tellurite) to their respective elemental species (Se^0^, Te^0^) (Montenegro et al., 2021; Vieto et al., 2021).

In this work, we have identified a collection of genes involved in selenite reduction in *P. putida* KT2440 through random mutagenesis, phenotypic analyses, determination of the selenite transformed and RNA sequencing. Our results indicate that selenite reduction takes place by the involvement of activities related to sulphur metabolism, central metabolic reactions and the resistance to oxidative stress. We have also identified activities that compete with selenite reduction, and shown that knock outs in the corresponding genes result in faster selenium nanoparticle formation, which could lead to biotechnological applications of *P. putida* for the detoxification and valorisation of selenite waste.

## EXPERIMENTAL PROCEDURES

### Bacterial strains, plasmids, culture media and growth conditions

*Pseudomonas putida* KT2440 was cultured aerobically in lysogeny broth (LB) broth at 30°C with orbital shaking at 150 rpm. *E. coli* strains were cultured in LB broth at 37°C with orbital shaking at 200 rpm. *E. coli* CC118λpir (pBAM1) (Martínez‐García et al., 2011) medium was supplemented with ampicillin (100 μg/ml) and kanamycin (50 μg/ml) while *E. coli* HB101 (pRK600) medium was supplemented with chloramphenicol (30 μg/mL). All chemicals were purchased from Sigma‐Aldrich.

### Mutagenesis with pBAM1

Mutagenesis of *P. putida* KT2440 by random insertion of miniTn5 transposons was conducted as described in (Martínez‐García et al., 2011). Briefly, pBAM was transferred to *P. putida* via triparental mating using *E. coli* CC118λpir (pBAM1) as a donor and *E. coli* HB101 (pRK600) as a helper. Each strain was grown independently overnight in LB broth and the cell pellet of 1 ml of each culture was washed with 1 ml of LB three times before resuspending in 100 μl of LB and mixed with the other strains. 100 μl of the mixture were deposited on a 0.22 μm nitrocellulose filter on a LB plate and incubated overnight at 30°C. After conjugation the cells on the filter were recovered by resuspending in 1 ml of LB and plated in selective media containing *Pseudomonas* Isolation Agar (Gifco), kanamycin and 1 mM sodium selenite. Plates were incubated at 30°C until colonies achieved an observable phenotype. The location of the Tn5 insertions was determined using arbitrary PCR of genomic DNA obtained from cell colonies as described previously (Das et al., 2005; Martínez‐García et al., 2011). PCR products were subject to Sanger sequencing and the resulting sequences were subject of blast (Altschul et al., 1990) against the *P. putida* KT2440 genome.

### Cloning of genes from *P. putida* KT2440 for complementation tests

For mutant complementation the protocol described by Montenegro et al. (2021) was used. Briefly the DNA sequence encoding the respective gene for each mutant (see Table 1) of *P. putida* KT2440 was amplified directly from the chromosome of the bacterium with their corresponding primers (see Table S3). The resulting amplicon was digested (see Table S3), and further ligated to the polylinker of the expression plasmid pSEVA438 as an either EcoRI/HindIII or BamHI/HindIII fragment (see Table S3) and subsequently transformed and propagated in *E. coli* DH5α. To verify the ligation, we replated colonies in fresh media with streptomycin (Sm 100 μg ml^−1^); a colony PCR was performed with the respective primers for each gene. After verifying the ligation of the plasmid we transformed the construct into the Se‐delayed mutants (See Table 1). Se‐delayed mutants were also transformed with the empty backbone (pSEVA438) as a control. To establish if the genetic complementation of the mutant was enough for acquiring the WT phenotype again, we grew preinocula of the mutants complemented and with the empty backbone (pSEVA438) overnight in Luria‐Bertani (LB) as described above. Optical density (OD) was measured and adjusted to 0.05 in fresh media supplemented with selenite (1 mM), Sm (100 μg ml^−1^) and 3‐methylbenzoate (3 MB, 0.5 mM). Strains were incubated with orbital shaking at 175 rpm at 30°C. Phenotype was analysed after 24 h of incubation.

**Table 1:** Genes mutated in *Pseudomonas putida* KT2440 by insertion of a kanamycin resistance cassette selected by phenotype.

Mutant ID	Locus tag	Gene name	Function of the disrupted gene	Metabolic pathway	Phenotype
RF30	PP_3999	*cysG*	Uroporphyrin‐III C‐methyltransferase	Sulphur metabolism/Porphyrins	Delayed
RF31	PP_3999	*cysG*	Uroporphyrin‐III C‐methyltransferase		
JJ3	PP_3999	*cysG*	Uroporphyrin‐III C‐methyltransferase		
JJ7	PP_2370		Hypothetical conserved protein	Sulphur metabolism	
JJ13	PP_0051	*sqrR*	Putative dependant sigma‐54 transcriptional regulator		Fast
JJ33	PP_0052	*pdo2*	Persulphide dioxygenase		
JJ14	PP_0053	*sqr*	Sulphide‐quinone oxidoreductase		
RF16	PP_4189	*sucA*	2‐oxoglutarate dehydrogenase	Central metabolism/Glutathione biosynthesis	Delayed
RF18	PP_5154	D2HGDH	D‐2‐hydroxyglutarate dehydrogenase		
RF20	PP_5154	D2HGDH	D‐2‐hydroxyglutarate dehydrogenase		
RF4	PP_3998	*gqr*	Glutathionyl‐hydroquinone reductase	Glutathione biosynthesis	
JJ24	PP_4322	*ccmF*	Holocytochrome *C* synthetase	Cytochrome *c* biosynthesis	Delayed
RF24	PP_4799	—	Muranoyltetrapeptide carboxypeptidase	Membrane structure	Delayed
RF29	PP_4935	*msbA*	lipid transporter ABC ATPase		
JJ5	PP_4936	*wzy*	O‐antigen polymerase		

### Growth curves of the mutants during selenite reduction

The effect of the mutations on *P. putida* KT2440 cultured in the presence or absence of selenite was assessed by monitoring growth kinetics at 600 nm (OD600) of the WT and mutants in 96 well microtiter plates (Surface Nunclon™) using a Sinergy H1 Hybrid Multi‐Mode microplate reader (BioTek). For this, cultures of the WT or each mutant were diluted to an initial optical density at 600 nm (OD600) of approximately 0.05 in fresh medium with 0 and 1 mM sodium selenite in a volume of 200 μl per well. Microplates were incubated at 30°C for 24 h with continuous orbital shaking and measurement of optical density every 10 min. Each growth curve consisted of three biological replicates. Photos of the plates were taken at 24 h of the experiment to record the coloration of the medium.

### Determination of colony‐forming units

*Pseudomonas putida* KT2440 and mutant strains were grown overnight in LB at 175 rpm and 30°C. These cultures were diluted to an initial OD600 of approximately 0.05 in fresh 25 ml of LB medium containing selenite (1 mM) and incubated in the same conditions for 24 h. Samples were taken each 2.5 h and each sample was serially diluted from 10^0^ to 10^−8^ in phosphate‐buffered saline (PBS) and plated onto Triptone soy agar (TSA) plates. Colonies were counted after 24 h of incubation at 30°C and the CFU ml^−1^ of three biological replicates were calculated.

### Determination of residual selenite amount by Flame Atomic Absorption Spectroscopy

Selenite reduction was recorded by analysis of residual selenite in the culture medium. For this, 3 L Erlenmeyer flasks with 500 ml of LB supplemented with 1 mM selenite were inoculated with *P. putida* KT2440 (initial OD of 0.05). The mutants were grown at 30°C and 150 rpm for 24 h. For Se‐delayed mutants, 15 ml aliquots of the cell suspension were taken at 0 h and 24 h during bacterial growth, while for mutants with the capacity to produce selenium more rapidly, samples were taken between 0 h and 20 h with intervals of 2.5 h. OD600 of samples was measured, and the cells were collected by centrifugation (10 min, 4000 *g*, 4°C). The supernatant was then carefully transferred to a new 15 ml tube and stored at −25°C until further analysis. For analytical determinations, supernatants were filtered on mixed cellulose ester syringe filters (0.20 μm; ADVANTEC®) and analysed using an atomic absorption spectrometer (AA240FS + 240Z; Varian Inc.). The wavelength (nm) used for selenium was 196.026. Selenite solutions were prepared from a 1000 mg/L standard solution (Tritrisol‐Merck). Appropriate dilutions were made to prepare the standards, which were stored in polyethylene flasks under refrigeration. The average of three replicates is presented in the bars and the error was calculated as the standard deviation of the results.

### RNA sequencing experiments

*Pseudomonas putida* KT2440 was precultured overnight in LB medium, and the culture was then diluted 100‐fold in the same medium and grown to OD600 of 0.2. Samples were then either cultured further without additional modifications or treated with 1 mM selenite for 1 h. Transcription in each culture was halted by spiking a high concentration of rifampicin (200 μg/ml). Total RNA was isolated from 1 ml of cells from each culture using a miRNeasy kit (Qiagen) with some modifications. The collected pellets were resuspended into 0.1 ml Tris–HCl (pH 7.5) containing lysozyme (2 mg/ml) and incubated for 10 min at 37°C. The lysate was processed according to manufacturer's instructions. RNase‐free DNase (Qiagen) treatment was performed during the RNA isolation procedure to eliminate residual DNA and the quality of isolated RNAs was evaluated using a 2100 Bioanalyzer System (Agilent). To obtain transcriptional profile at the genome‐wide level, libraries of cDNA with an average size of 200 nucleotides obtained from two independent replicates per condition were sequenced using a HiSeq 2000 platform (Illumina) by Novogene Corporation. Raw data (fastq files) was uploaded to the NCBI's database GEO with accession number GSE214391.

The DESeq2 package version 1.34.0 was used for normalization and differential expression analysis between control and treatment samples following mapping and counting, with effect size shrinkage using the ‘apeglm’ approach (Zhu et al., 2019) (Data can be accessed in Supplementary Files [Data S1 and S2] ‘Selenite.RawCounts.csv’ and ‘FPKM_log2_All.csv’; reproducibility between experiments is shown in Figure S15). Gene annotation was carried out using the tool of the DESeq2 package and the appropriate file (gtf) from the assembly (GCA_000014625.1). Genes with a log‐Fold Change >2 and adjusted *p*‐value <0.05 were considered differentially expressed (DEGs). DEGs were functionally classified using the goseq R package version 1.46 (Young et al., 2010) and the Kyoto Encyclopedia of Genes and Genomes (KEGG) database (http://www.genome.jp/kegg/) (Kanehisa & Goto, 2000). GO annotation and KEGG classifications were downloaded from the *Pseudomonas* Community Annotation Project (PseudoCAP).

## RESULTS

### Phenotypes and growth rates of mutants involved in selenite reduction

**FIGURE 2:**
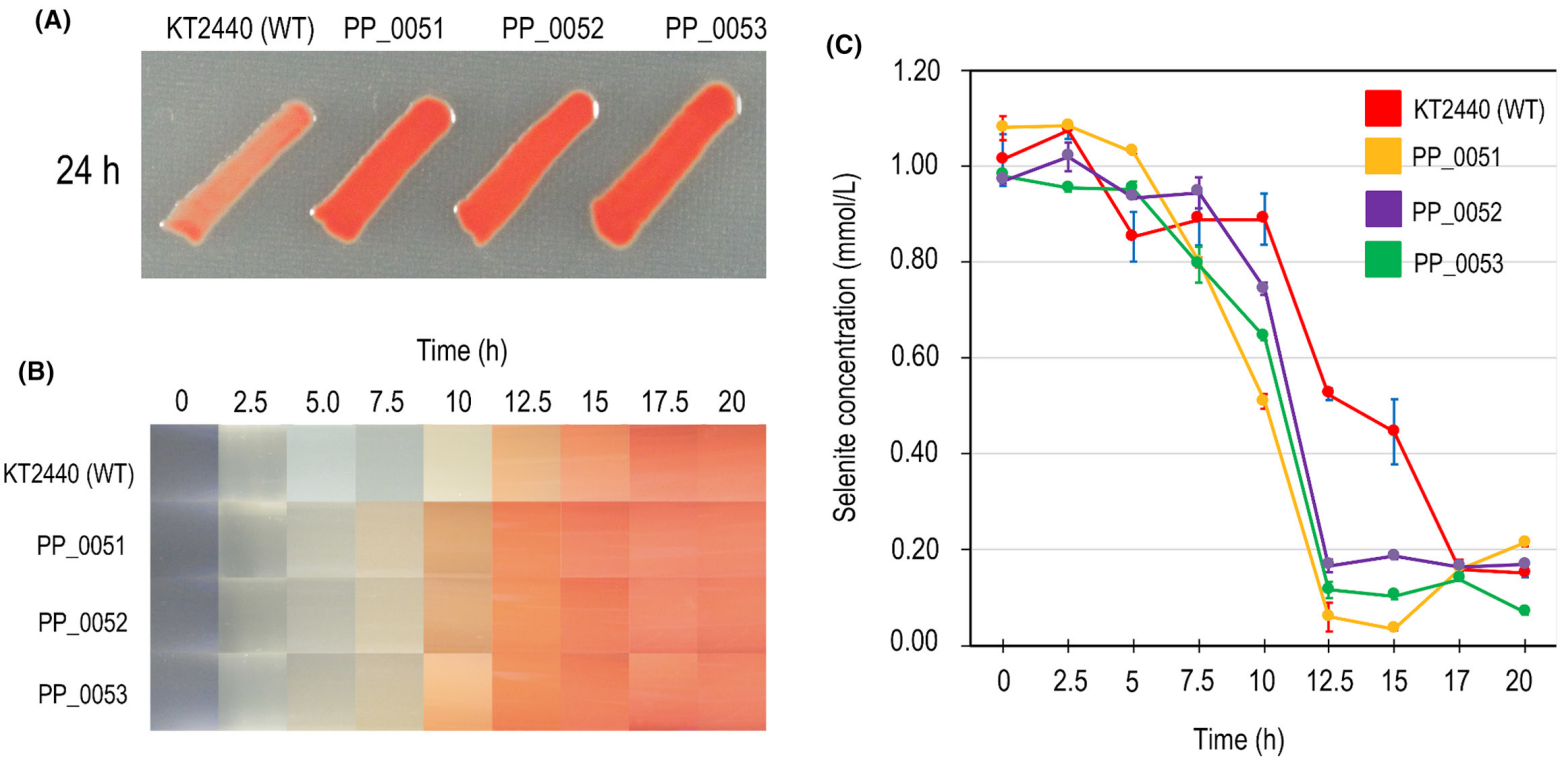
Growth of *Pseudomonas putida* KT2440 and fast mutants in lysogeny broth (LB) broth in the presence of selenite (1 mM). (A) Photographs of *P. putida* KT2440 (WT) in solid LB medium in the presence of selenite. The photographs were taken after 24 h of growth. (B) Heat map of selenite reduction in *P. putida* KT2440 and fast mutants. The heat map was made using the photographs from Video S1. (C) Kinetics of selenite reduction in *P. putida* KT2440 and fast mutants measured by Flame Atomic Absorption Spectroscopy during 20 h. As can be seen both in the photographs and in the reduction kinetics, the PP_0051, PP_0052 and PP_0053 mutants reduce selenite more quickly.

A total of 15 mutants related to selenium metabolism were obtained (Table 1). Twelve mutants (in genes *cysG*, PP_2370, *sucA*, *D2HGDH*, *gqr*, *ccmF*, *ldcA*, *msbA* and *wzy*) had a phenotype delayed in the production of elemental selenium nanoparticles, meaning that the colonies had a white to pink colour during the first 24 h when cultured in LB medium with 1 mM selenite (Figure 1B). This lack of reddish coloration denotes that these mutants cannot generate elemental selenium at the same rate or yield as *P. putida* KT2440. In the remainder of the manuscript, we will refer to these mutants as ‘Se‐delayed mutants’. The rest of the mutants (*sqrR*, *sqr* and *pdo2*) were capable of producing selenium at a higher rate than the wild‐type (WT) strain (Figure 2A, B and Video S1). When observed in an LB culture with 1 mM selenite, they turned reddish noticeably faster than the WT strain. In the remainder of the manuscript, we will refer to these mutants as ‘fast mutants’.

The growth of the Se‐delayed mutants in LB medium without selenite showed in all cases a growth rate similar to the WT strain except for the *sucA* mutant (PP_4189), which showed a very slight reduction in the growth rate and the formation of biomass (Figure S1A). This phenotype is not surprising since *sucA* encodes the enzyme 2‐oxoglutarate dehydrogenase, a key enzyme in the Krebs cycle. In the presence of selenite, three mutants appear to have a slower growth rate than the WT strain, including again the *sucA* mutant. The other two mutants correspond to disruptions in genes *ccmF* (encoding a holocytochrome *C* synthetase) and *wzy* (enconding an O‐antigen polymerase). In the presence of selenite, the final optical density was highly variable among mutants (Figure S1B). This phenomenon occurs because elemental selenium also absorbs or scatters radiation at 600 nm and the values measured correspond to cell growth and variable amounts of elemental selenium. We discerned the specific effect of mutations on cell growth determining colony‐forming units (CFU) of all strains in the presence of 1 mM selenite (Figure S2A). The results showed that there are no differences in CFUs between the WT strain and the mutants for at least 22 h of growth, hence variations observed in absorbance at 600 nm were due to the formation of selenium nanoparticles. As mentioned above, three mutants showed a very noticeable phenotype in Petri dishes and liquid cultures: these mutants turned reddish faster than *P. putida* KT2440 (Figure 2A,B and Video S1). These fast mutants were affected in the genes *sqrR* (encoding a putative dependent sigma‐54 transcriptional regulator), *sqr* (encoding a sulphide‐quinone oxidoreductase) and *pdo2* (encoding a persulphide dioxygenase). Growth curves of these mutants in LB medium (Figure S3A) indicated that their growth rate was similar to the WT strain. In the presence of selenite, a rapid increase in optical density was observed at 600 nm due to the rapid production of selenium nanoparticles (Figure S3B). This effect began to be visible after 4 h for the three mutants. It is clear that the difference in the optical density observed in Figure S3B was a product of the absorbance or dispersion of the selenium nanoparticles and not due to the accelerated growth of the mutants since in the absence of selenite (Figure S3A), no changes were observed in the growth of these mutants compared to the WT. As mentioned above, CFU kinetics (Figure S2B) showed no differences in growth between the WT and the three fast mutants, suggesting that the mutations in the *sqrR*, *sqr* and *pdo2* genes resulted in an accelerated production of selenium nanoparticles and not in an increase in growth rates.

The reduction of selenite to elemental selenium in the mutants was monitored by determining the amount of selenite remaining in LB cultures using Flame Atomic Absorption Spectroscopy (FAAS). For mutants delayed in selenite reduction, the presence of this oxyanion in the culture medium was determined after 24 h of growth. As expected, all Se‐delayed mutants had lower bioconversion values than the WT strain (Figure 1C). However, results suggest that none of the mutated genes was essential for this process since (i) in all the cases it was possible to observe some bioconversion after 24 h (Figure 1C) and (ii) after 48 h of growth, all of these mutants manged to reach a reddish coloration almost of the same intensity as the WT (Figure S4), suggesting that there is more than one pathway to reduce selenite. Therefore, it is important to clarify that the term delayed does not denote a complete inability to metabolize selenite but rather denotes a limited ability to reduce selenite at the same rate as the WT strain.

To corroborate the differences in the conversion rate of selenite between mutants *sqrR*, *sqr* and *pdo2* compared to the WT strain, we determined in LB cultures enriched with 1 mM selenite the remaining content of oxyanion in the culture medium between 0–20 h of growth at intervals of 2.5 h (Figure 2C). Results showed that mutants *sqrR*, *sqr* and *pdo2* started the reduction process several hours earlier than the WT strain; however, the yield of selenium nanoparticles was the same in all cases (WT and mutants) after 17.5 h (Figure 2B, C and Video S1). In our previous study (Avendaño et al., 2016), we concluded that the selenite reduction process in *P. putida* KT2440 begins in the middle‐exponential phase; however, in *sqrR*, *sqr* and *pdo2* mutants, the reduction is activated at the beginning of the exponential phase.

Using the data from Figure 2C, we determined the rate of transformation of selenite in mmol ${\mathrm{SeO}}_{3}^{-2}\;L^{-1}\;h^{-1}{\mathrm{OD}}_{600}^{-1}$. For this calculation, a linear model was assumed during biotransformation and a zero order of reaction. Furthermore, the concentration values were normalized with respect to the optical density. With this estimation, a transformation speed was obtained for the WT strain of 0.086 ± 0.020 mmol ${\mathrm{SeO}}_{3}^{-2}\;L^{-1}\;h^{-1}{\mathrm{OD}}_{600}^{-1}$, while for *sqrR*, *sqr* and *pdo2* mutants, speeds between 2.2 and 3.6 times higher were obtained (0.307 ± 0.067 mmol ${\mathrm{SeO}}_{3}^{-2}\;L^{-1}\;h^{-1}{\mathrm{OD}}_{600}^{-1}$, 0.197 ± 0.025 mmol ${\mathrm{SeO}}_{3}^{-2}\;L^{-1}\;h^{-1}{\mathrm{OD}}_{600}^{-1}$ and 0.193 ± 0.021 mmol ${\mathrm{SeO}}_{3}^{-2}\;L^{-1}\;h^{-1}{\mathrm{OD}}_{600}^{-1}$, respectively).

### Complementation experiments in trans

In order to validate whether the observed differences in selenium formation were due to the absence of each gene individually, we ran complementation tests. For these experiments, we constructed plasmids using the pSEVA438 plasmid (Silva‐Rocha et al., 2013) in which genes *cysG*, *sucA*, *D2HGDH*, *gqr*, *ccmF*, *ldcA*, *msbA* and *wzy* were individually expressed under the control of a promoter inducible with 3‐methylbenzoate (3 MB; see Experimental Procedures). These plasmids were introduced within the corresponding mutant, that is, each mutant was complemented with its respective gene in trans. For example, for the *cysG* mutant, a plasmid pSEVA438‐*cysG* was constructed, electroporated and the gene expressed after induction with 3 MB. As seen in Figure S5, mutants with the empty vector (i.e. pSEVA438) maintain the delayed phenotype in selenite reduction (first row in the figure). However, when the mutants were complemented with the respective gene cloned in the plasmid and induced with 3 MB, the selenite reduction was restored to the levels of the WT strain (second row in Figure S5). These results validate those shown in Figure 1B,C and demonstrate the involvement of these genes in selenite metabolism in *P. putida* KT2440.

### Transcriptional analysis of WT *P. putida* KT2440 in the absence and presence of selenite

We investigated the global transcriptional response to selenite of *P. putida* KT2440 preparing RNAseq libraries of cultures grown in the presence of the oxyanion. We extracted the RNA from cultures growing on LB in the exponential phase in the absence of selenite as a control or exposed to 1 mM selenite for 1 h. Libraries were prepared from two independent biological replicates in each case. We obtained an average of 17.6 million reads per sample, and after the removal of low‐quality reads, 93.7% of reads were mapped to the genome of *P. putida* KT2440 (NC_002947.4) (Belda et al., 2016). Reads aligned with most open reading frames except PP_1099, PP_1149 and PP_2463.

**FIGURE 3:**
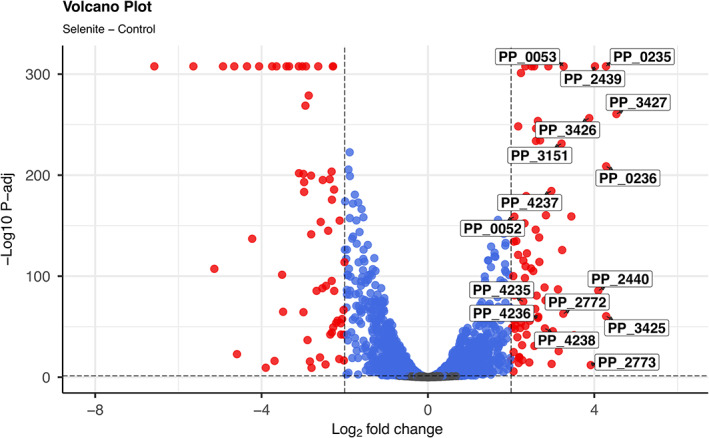
Transcriptional changes in the presence of selenite. Differential gene expression relative to control treatment is shown in the presence of selenite. Each point shows one gene. Red points show significantly differentially expressed genes (*p*‐adjusted <0.05 and log2 fold change >2). Labels correspond to genes discussed in the main text.

**FIGURE 4:**
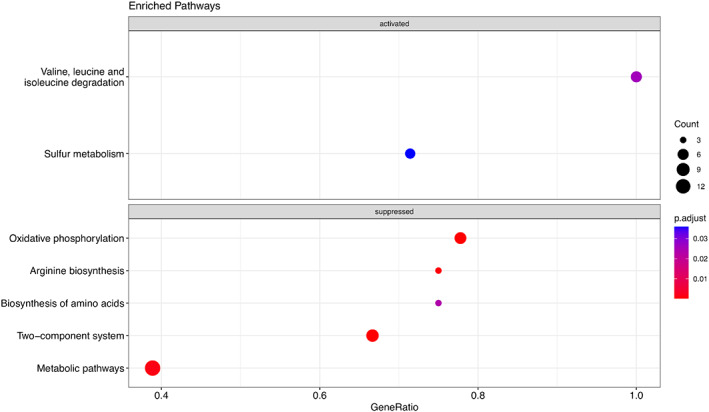
Enrichment pathways using KEGG database. Enrichment analysis was conducted comparing differentially expressed genes against the KEGG *P. putida* KT2440 database. Gene ratio corresponds to the fraction of differentially expressed genes in pathways.

**FIGURE 5:**
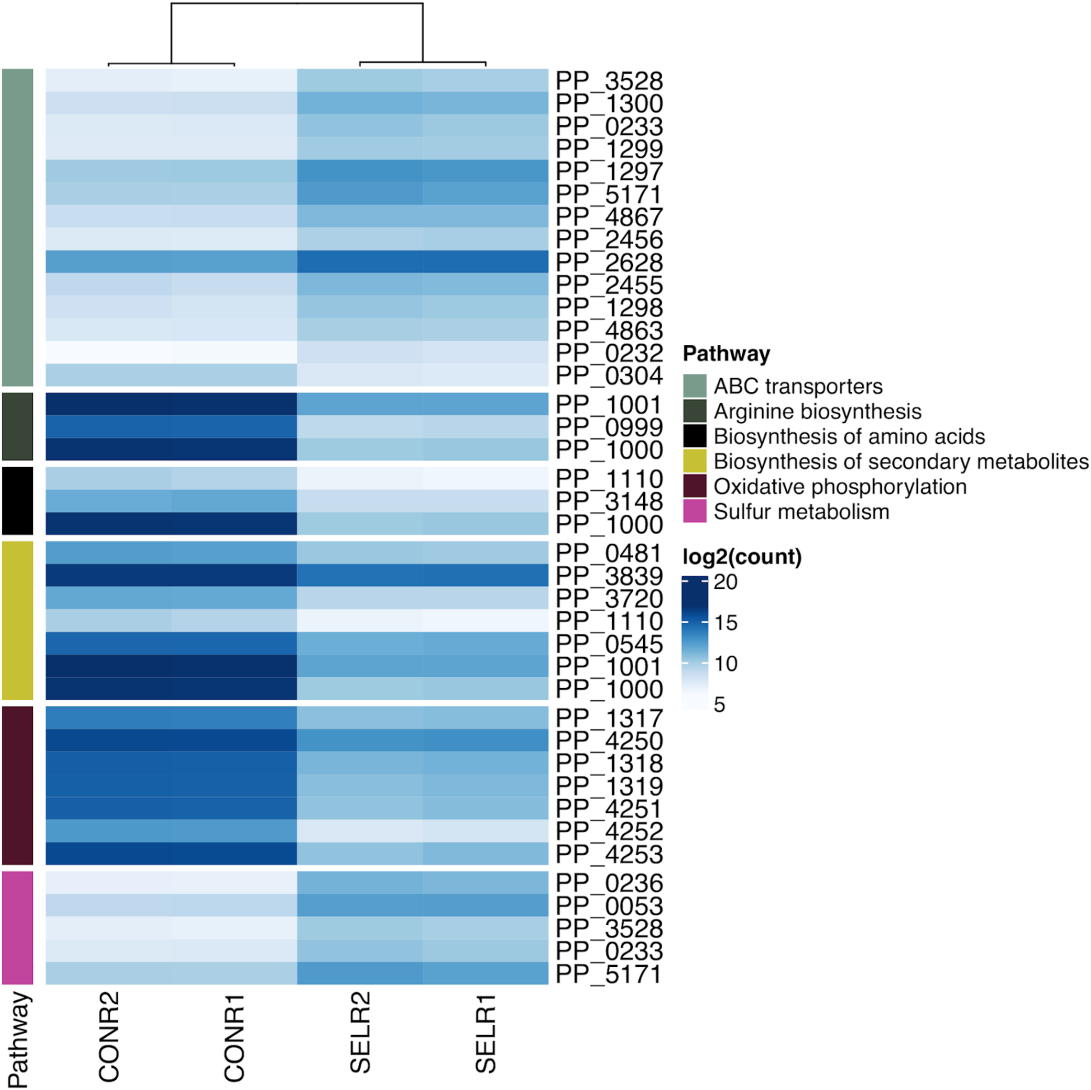
Heatmap representing selected pathways differentially expressed in the presence of selenite.

Many genes were differentially expressed in response to exposure to selenite (Figure 3). They were classified according to their functional categories using the COG database (Figure 4; Tables S1 and S2). We detected overexpression of 2 functional categories while 7 functional categories were underrepresented in the presence of selenite. Several functions were enriched in each condition (Figure 5; corresponding KEGG pathways are shown in Figures [Link], [Link]). Exposure to selenite triggered the expression of transporters (or more broadly membrane associated proteins) and genes related to sulphur metabolism. The exposure to selenite led to the underexpression of metabolic functions involved in the metabolism of amino acids and oxidative phosphorylation. Again, the synthesis of extracellular selenium could have an impact on the membrane bound respiratory chain, preventing its correct functioning, which would affect the cellular metabolism as a whole. Moreover, the stress generated by selenite is likely to also affect metabolic processes.

When investigating specific genes, the expression profile was consistent with a response to mitigate oxidative stress (see Supplementary file FPKM_log2_All.csv). The most highly expressed genes included PP_3427, PP_3425 and PP_3426 encoding the multidrug efflux RND outer membrane protein OprN and the multidrug efflux RND transporter MexEF, which have been reported to be overexpressed in *P. putida* during the detoxification of formaldehyde (Roca et al., 2008). Other highly expressed genes in presence of selenite encode the peroxidase LsfA, the alkyl hydroperoxide reductase AhpCF (PP_2439 and PP_2440) and the semialdehyde dehydrogenase SadI (PP_3151). Some of the overexpressed genes involved in sulphur metabolism were the NAD(P)H‐dependent FMN alkanesulfonate monooxygenase SsuEF (PP_0235 and PP_0236), the putative dimethyl sulfone monooxygenase SfnCE (PP_2772 and PP_2773) and the disulphide bond forming complex Dsb (PP_4235, PP_4236, PP_4237 and PP_4238). Of the genes identified in the mutagenesis screening, only *sqr* and *pdo2* (PP_0052 and PP_0053), together with the last gene in the operon they form (PP_0054) were significantly overexpressed in response to selenite (Figure 3). These genes are responsible for the fast phenotype when disrupted, which delay selenite reduction in the WT strain as their mutation leads to increase the rate of selenium production.

## DISCUSSION

### Genes related to 2‐ketoglutarate and glutamate metabolism participate in selenite reduction

**FIGURE 6:**
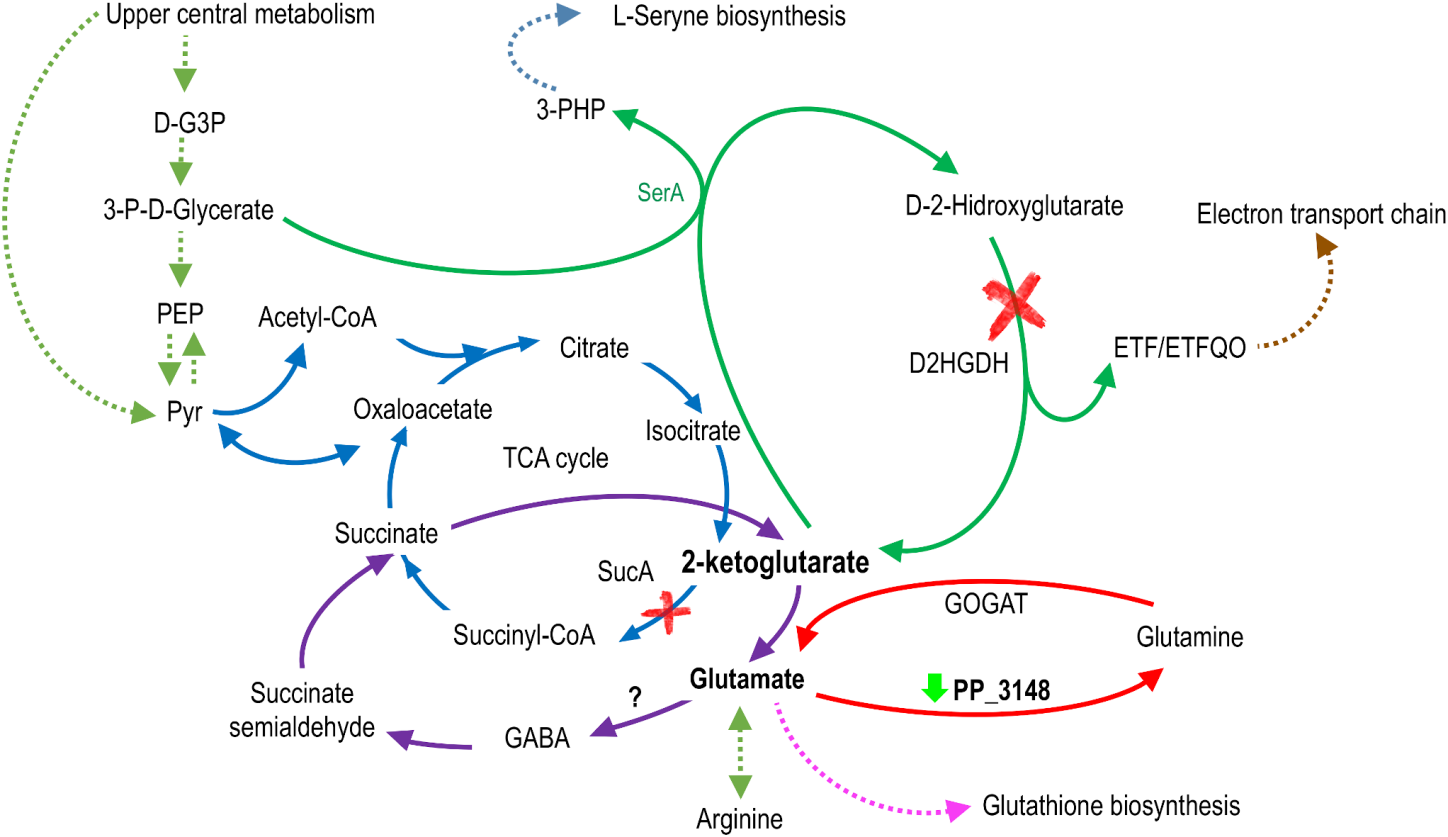
Reduced representation of 2‐ketoglutarate and glutamate metabolism in *Pseudomonas putida* KT2440. The mutation in the genes that encode the enzymes SucA and D2HGDH is represented with a red ‘X’. Mutation in these genes showed a delayed phenotype in selenite reduction. Both mutants correspond to enzymes related to the metabolism of 2‐ketoglutarate, a key metabolite that is part of the Krebs cycle (blue) and is the starting point for the synthesis of glutamate and glutathione. Furthermore, recent studies connect this metabolite with serine synthesis (green). An alternative route for the synthesis of succinate (purple) that is not through the enzyme SucA is also represented in the figure. In the transcriptomic analysis, genes that were differentially expressed in the presence of selenite were observed. One of them is PP_3148 that was underexpressed (downward green arrow) and encodes a glutamine synthetase.

*Pseudomonas putida* possesses a robust central metabolism capable of reconfiguring the metabolic fluxes towards NADPH‐producing reactions such as those catalysed by the enzymes Zwf and GntZ (Nikel et al., 2021). This cofactor is necessary for the action of the enzyme glutathione reductase (Gor, PP_3819) and to maintain glutathione in its reduced form and thus fight against oxidative stress. Since glutathione is a key molecule, it is presumable that any changes in its biosynthesis may alter the cellular capacity to resist oxidative stress and carry out reduction processes. Mutagenesis with Tn5 transposons combined with a screening in the presence of selenite allowed to obtain two mutants (*sucA* and *D2HGDH*) delayed in the reduction of selenite which, according to their metabolic functions, affect the 2‐ketoglutarate pool, a master regulator metabolite (Huergo & Dixon, 2015) and the starting point for glutamate and then glutathione biosynthesis. The first mutant had inactivated the *sucA* gene encoding the E1 subunit of 2‐ketoglutarate dehydrogenase. This enzyme is responsible for transforming 2‐ketoglutarate to succinyl‐CoA in the Krebs cycle, so it could be presumed that *sucA* is an essential gene. However, as seen in Figure S1, this mutant grew very similarly to the WT strain, what could be explained in different ways. First of all, our experiments were performed in LB rich medium, which can provide various carbon sources and synthesize essential metabolites from different metabolic steps. For example, Zhang et al. (2018) demonstrated that *P. putida* KT2440 could grow with L‐lysine as a carbon source using glutamate as an intermediate and a combination of enzymes that can transform 2‐ketoglutarate directly to succinate, that is, not via *sucA*. Secondly, in the absence of 2‐ketoglutarate dehydrogenase, an alternative pathway to the Krebs cycle has been reported in several bacterial species (Green et al., 2000; Xiong et al., 2014); therefore, we hypothesized that in *P. putida* a similar cycle with glutamate, gamma‐aminobutyric acid (GABA) and succinate semialdehyde could also be operating (Figure 6). According to the KEGG Pathway Database, in the genome of *P. putida* KT2440 the enzymes responsible for transforming GABA to succinate semialdehyde (PP_0214 and PP_2799) and succinate semialdehyde succinate (PP_0213, PP_2488, PP_3151 and PP_4422) are present. However, an enzyme with glutamate decarboxylase activity responsible for transforming glutamate to GABA has not been described. Although there is no annotated enzyme for this reaction in the literature, it has been described that several species of *Pseudomonas* are capable of synthesizing GABA (Dagorn et al., 2013). In the context of this work, we consider that in a *sucA* mutant, an alternative pathway could be present (represented in purple in Figure 6), causing a reduction in glutamate availability, which, in turn, could affect glutathione synthesis. In the WT strain glutamate synthesis is carried out from 2‐ketoglutarate of the tricarboxylic acid cycle, serine synthesis or proline degradation (Gryder & Adams, 1969; Revelles et al., 2005; Zhang et al., 2016) and the pool of glutamate is used for biosynthesis of glutamine, glutathione and porphyrins such as cytochrome *c* and siroheme. However, in the *sucA* mutant according to the model shown in Figure 6, 2‐ketoglutarate and glutamate metabolism would require a reconfiguration of the metabolic network. Interestingly, in the transcriptomic experiments the genes involved in the transformation from 2‐ketoglutarate to Succinyl‐Co‐A (i.e. *sucA*, *sucB* and *lpdA*), were underexpressed in the presence of selenite (Figure S11), which supports the results obtained in the mutagenesis. Other central metabolic genes such as those encoding the succinate dehydrogenase and the pyruvate carboxylase (Figure S11) were also underexpressed, which supports the idea that the Krebs cycle and specifically 2‐oxoglutarate is involved in the reduction of selenite. It is possible that the presence of selenite generates oxidative stress affecting the pathways involved in glutathione synthesis, specifically in 2‐oxoglutarate and glutamate metabolism. Underexpression of gene PP_3148 in transcriptomic analysis (Figures 5 and 6), also supports this hypothesis. PP_3148 encodes a glutamine synthetase responsible for the transformation of glutamate to glutamine, which again reaffirms the participation of 2‐oxoglutarate and glutamate and their metabolism in selenite reduction. Interestingly, none of the genes with GOs related to glutathione metabolism (i.e. coding for enzymes using glutathione as cofactor) changed their expression levels in response to selenite.

A mutant in the D‐2‐hydroxyglutarate dehydrogenase (D2HGDH) showed a delayed phenotype in the selenite reduction process (Figure 6). We hypothesize that this mutation also affects the pool of 2‐ketoglutarate and glutamate. D2HGDH catalyses the reaction that converts D‐2‐hydroxyglutarate (D‐2‐HG) to 2‐ketoglutarate. D‐2‐HG is a key player in the central metabolism of *Pseudomonas* including *P. putida* KT2440 (Zhang et al., 2016). In these bacteria, the synthesis of L‐serine is initiated by the enzyme SerA, which couples the dehydrogenation of D‐3‐phosphoglycerate to 3‐phosphohydroxypyruvate and the reduction 2‐ketoglutarate to D‐2‐HG (Zhang et al., 2016) (see Figure 6). Subsequently, 2‐ketoglutarate is replenished from D‐2‐HG thanks to the D2HGDH activity (Zhang et al., 2016). We hypothesize that by inactivating the enzyme D2HGDH, 2‐ketoglutarate cannot be replaced from D‐2‐HG, affecting the pool of 2‐ketoglutarate and glutamate and as a consequence the biosynthesis of glutathione is also affected. This mutant not only allows us to strengthen our conclusions about the importance of 2‐ketoglutarate and glutamate metabolism in selenite reduction but also allows us to make a connection with the synthesis of other amino acids, such as serine. (Figure 6). The transcriptomic analysis also evidenced variations in the expression of genes related to the metabolism of serine. The PP_1110 gene, underexpressed in the presence of selenite, encodes a serine acetyltransferase, an enzyme that links amino acid and sulphur metabolism.

The differential expression of genes related to the biosynthesis of arginine obtained in the presence of selenite in the RNA sequencing experiments (Figures 4 and 5, Figure S7) also points to the participation of the metabolism of 2‐oxoglutarate and glutamate in the reduction of selenite since the biosynthesis of arginine is carried out from glutamate or glutamine. As seen in Figure 5 and Figure S7, in addition to glutamine synthetase (PP_3148; EC: 6.3.1.2), a carbamate kinase (PP_0999; EC: 2.7.2.2), an ornithine carbamoyl transferase (PP_1000; EC: 2.1.3.3), and an arginine deiminase (PP_1001; EC:3.5.3.6) involved in arginine biosynthesis were underexpressed in the presence of selenite.

### The response to oxidative stress is activated in the presence of selenite

**FIGURE 7:**
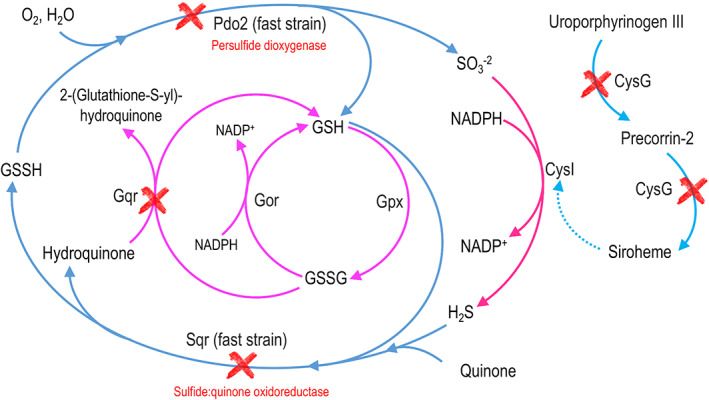
Reduced representation of sulphite and glutathione metabolism in *Pseudomonas putida* KT2440. The genes that were truncated in the mutagenesis experiments are marked with a red ‘X’. Mutations in genes *cysG* and *gqr* showed a delayed phenotype, while mutations in genes *sqr* and *pdo2* showed a fast phenotype in selenite reduction. Fast mutants were truncated in genes that recycle H_2_S (and maybe H_2_Se) to ${\mathrm{SO}}_{3}^{-2}$ (and maybe ${\mathrm{SeO}}_{3}^{-2}$), suggesting that sulphide:quinone oxidoreductase and persulphide dioxygenase constitute a pathway that competes with the reduction of selenite to elemental selenium. On the other hand, the mutation in *cysG* suggests an impairment in the activity of sulphite reductase CysI. A mutant in the PP_2370 gene (which forms an operon with *cysI*) was also obtained during mutagenesis, reinforcing the idea that the sulphite reductase activity is important for selenite reduction. The mutation in *gqr* also indicates the importance of glutathione metabolism in selenite reduction.

The mutagenesis experiments allowed obtaining a Se‐delayed mutant with a truncated glutathionyl‐hydroquinone reductase (Gqr) gene. This class of enzymes are widely distributed in bacteria, halobacteria, fungi and plants and catalyse the reduction of glutathionyl‐hydroquinones to hydroquinones (Green et al., 2012). This enzyme belongs to the superfamily of glutathione transferases that are involved in cellular detoxification against harmful xenobiotics and endobiotics (Allocati et al., 2009). This reaction contributes to the regeneration of GSH (reduced glutathione) from GSSG (oxidized glutathione) (Figure 7), which is undoubtedly involved in the oxidative stress response. In the Gqr mutant this replenishment is blocked, possibly directly impacting the GSH pool and its oxidative stress response. According to the functions described in the literature, there is no doubt that Gqr is essential for the cellular response to oxidative stress generated by selenite in *P. putida*.

The transcriptomic analysis also reflected the overexpression of other genes related to the response to oxidative stress namely the peroxidase LsfA, the alkyl hydroperoxide reductase AhpCF (PP_2439 and PP_2440) and the semialdehyde dehydrogenase SadI (PP_3151) (Figure 3). Other genes related to oxidative stress such as those coding for the cytoplasmic catalases KatA (PP_0481), KatE (PP_0115) and KatG (PP_3668) were not differentially expressed. Kaihami et al. (2014) reported that LsfA from the opportunistic pathogen *Pseudomonas aeruginosa* is endowed with thiol‐dependent peroxidase activity that protects the bacteria from H_2_O_2_. In *Pseudomonas fluorescens* it was also shown that this gene encodes an enzyme that has peroxidase and thioredoxin activity (Liu et al., 2015). Moreover, the alkyl hydroperoxide reductase AhpCF proved to be critical for survival of biofilm bacteria in *P. aeruginosa* in the presence of hydrogen peroxide (Panmanee & Hassett, 2009). The importance of AhpCF in resistance to oxidative stress has been well documented in several bacterial genera (Feng et al., 2020; Rocha & Smith, 1999; Wan et al., 2019). Therefore, the overexpression of the genes encoding LsfA and AhpCF in the presence of selenite, confirms that this oxyanion induces this component of the response to oxidative stress in *P. putida*.

### Sulphur metabolism is involved in selenite reduction

The non‐specific activity of some oxidoreductases on selenite is a controversial issue. It has been suggested that due to the structural similarity and chemical reactivity of species such as ${\mathrm{SO}}_{3}^{-2}$ and ${\mathrm{SeO}}_{3}^{-2}$, some enzymes can act on them in a non‐specific way. In the case of selenite, due to the proximity in the periodic table of sulphur and selenium, it is presumable that enzymes such as sulphite reductase (responsible for transforming sulphite to hydrogen sulphide; see Figure 7) can recognize selenite. In species of *Alcaligenes faecalis*, it was reported that selenite reduction occurs through the activity of proteins such as sulphite reductase and thioredoxin reductase (Wang et al., 2018). Moreover, the link between Se and S has also been shown in *Shewanella* sp. O23S, which is able to reduce ${\mathrm{SeO}}_{4}^{2-}$ and ${\mathrm{SeO}}_{3}^{2-}$ to Se^0^ particles with variable S content (Staicu et al., 2022). Our results indicate that sulphur metabolism is important for selenite reduction in *P. putida* KT2440. We isolated four mutants whose disrupted genes are related to sulphite reductase activity (mutants ID RF30, RF31, JJ3 and JJ7; Table 1). These mutants contained insertions in PP_2370 (unknown protein forming an operon with *cysI* encoding the Heme protein sulphite reductase, beta subunit) and *cysG* genes (PP_3999, encodes a Uroporphyrin‐III C‐methyltransferase) (Table 1). It is important to highlight that in the screening we isolated three mutants affected in *cysG* (Table 1, mutants ID RF30, RF31 and JJ3), which suggests that this gene is crucial for selenite reduction. It has previously been reported in *E. coli* that the sulphite reductase requires siroheme as a cofactor (Wu et al., 1991). Therefore, we hypothesize that the activity of the uroporphyrin‐III C‐methyltransferase is required for the synthesis of siroheme, a cofactor required for the action of the product encoded by *cysI*. Then, the hydrogen selenide produced by the activity of CysI may react with ambient oxygen in an abiotic manner, generating elemental selenium and superoxide as described in (Kessi & Hanselmann, 2004). We propose that the *cysI‐cysG* system performs at least partially the reduction of selenite to hydrogen selenide, following the pathway represented in Figure 7 for sulphur metabolism.

The involvement of sulphur metabolism in selenite reduction is supported by two other mutants obtained in this study: *sqr* (PP_0053), which encodes a sulphide‐quinone oxidoreductase and *pdo2*, which encodes a persulphide dioxygenase and whose function already has been experimentally demonstrated (Sattler et al., 2015; Shibata & Kobayashi, 2006). It is worth noting that we also isolated a mutant in the gene encoding for the putative transcriptional activator of the operon containing these genes (PP_0051). As shown in figure 7, the Sqr/Pdo2 system is responsible for recycling part of the H_2_S (and maybe H_2_Se) to ${\mathrm{SO}}_{3}^{-2}$ (and maybe ${\mathrm{SeO}}_{3}^{-2}$). The Sqr/Pdo2 system, which is opposed to the activity of the sulphite reductase, has been proposed as a mechanism responsible for maintaining the concentration of H_2_S (and maybe H_2_Se) in equilibrium between its formation and oxidation (Sattler et al., 2015). Consistent with this statement, Xia et al. (2017) suggested that the Sqr/Pdo system oxidizes the self‐produced sulphide to prevent its accumulation and volatilization as H_2_S. In the first step (catalysed by Sqr, Figure 7), the use of H_2_S is coupled with quinone and GSH to produce GSSH and hydroquinone. The sulphide‐quinone oxidoreductase activity of Sqr has been described in *P. putida* KT2440 (Shibata & Kobayashi, 2006) and its deletion led to a decrease in the intracellular catalase and ubiquinone‐H2 oxidase activity. In the second step, the enzyme Pdo2 oxidizes glutathione persulphide (GSSH) to sulphite and GSH (Sattler et al., 2015). Considering these two metabolic steps and the analogies between the chemistry of sulphur and selenium, it is reasonable to think that in the presence of selenite, the Sqr/Pdo2 system contributes to the maintenance of optimal H_2_Se levels. The overexpression of these genes in the presence of selenite suggests that processes, which may be considered antagonistic – selenite reduction and its prevention by, respectively, delayed and fast genes – act at the same time, possibly to ensure the appropriate balance of activities and usage of cofactors. As shown in Figure 2 and Video S1, the *sqr* and *pdo2* mutants metabolize selenite more rapidly than the WT, which suggests that blocking these two metabolic steps increases the availability of H_2_Se for its subsequent reduction to Se^0^. During this cyclical process (${\mathrm{SeO}}_{3}^{-2}$➔H_2_Se➔${\mathrm{SeO}}_{3}^{-2}$) mediated by Sqr/Pdo2, there is no net consumption of GSH. The identification of the enzymes Sqr and Pdo2 as targets to increase the production rate of selenium nanoparticles is of interest for the development of biotechnological tools.

The participation of sulphur metabolism in selenite reduction was also evidenced in transcriptomic experiments. Several genes related to sulphur metabolism were overexpressed including the NAD(P)H‐dependent FMN alkane sulfonate monooxygenase SsuEF, the putative dimethyl sulfone monooxygenase SfnCE and the disulphide bond forming complex Dsb (Figure 5 and Figure S6). The involvement of genes related to disulphide bond formation is consistent with a previously proposed pathway for the biological reduction of selenite *in E. coli* (Kessi & Hanselmann, 2004). In this model, a sequence of transformations including the formation of molecules with S‐Se bonds is suggested. Transcriptomic experiments also revealed that exposure to selenite triggered the expression of membrane associated proteins related to sulphur (Figure 5). In the presence of selenite, it was possible to detect the overexpression of transporters related to the uptake of sulphate (PP_5171) and the sulphur compound taurine (PP_0232, PP_0233) (Figure 5 and Figure S6). Previous studies provided biochemical evidence supporting the involvement of a sulphate transporter in the transport of selenite and selenate in *E. coli* K‐12 (Lindblow‐Kull et al., 1985). Lusa et al. (2017) studied with a proteomic approach the putative selenite transport systems in two environmental isolates of *Pseudomonas*. The authors suggest two different transport mechanisms for Se(IV) uptake in these *Pseudomonas* sp. strains: a low affinity transport system up‐regulated by ${\mathrm{NO}}_{3}^{-}/{\mathrm{NO}}_{2}^{-}/{\mathrm{SO}}_{4}^{2-}$ and a distinct ${\mathrm{SeO}}_{3}^{2-}$ regulated transport system. Considering this information, it is presumable that the transporter PP_5171 may be involved in selenite uptake in *P. putida* KT2440, reaffirming the importance of sulphur metabolism and including transport systems.

### Other genes involved in selenite reduction

In the mutagenesis experiments, a Se‐delayed mutant was obtained with an insertion in the gene *ccmF*, which encodes a holocytochrome *C* synthetase and is part of the well‐known cytochrome *c* maturation system. All the components of the Ccm system are located in the cytoplasmic membrane, which is responsible for the covalent attachment of haem to the CXXCH motif of apocytochrome in the periplasm (Cianciotto et al., 2005). Mutations in genes of the Ccm system have shown a great variety of phenotypes in bacteria, some of them with opposite effects. For example, in *P. fluorescens* (Gaballa et al., 1996, 1998), *P. aeruginosa* (de Chial et al., 2003) and other bacterial genera (Pearce et al., 1998), mutations in *ccm* genes resulted in reduced production and uptake of siderophores. However, in *P. putida* GB‐1, a reduction in pyoverdine production was not observed in different *ccmF* mutants (de Vrind et al., 1998). This study demonstrated that in *P. putida* GB‐1 mutants in *ccmF* did not have cytochrome oxidase activity, did not contain c‐type cytochromes and were deficient in the oxidation of Mn^2+^. Cianciotto et al. (2005) proposed that low levels of cytosolic haem are produced in *ccm* mutants, which prevents the maturation of several enzymes, including those involved in the biosynthesis of siderophores or in the resistance to oxidative stress. This relationship between the deficiency of the *ccm* genes and the impairment of the response to oxidative stress could explain the phenotype observed in the mutant *ccmF* concerning the reduction of selenite. Transcriptomic analysis also revealed that the presence of selenite led to the underexpression of two operons related to cytochrome activity and oxidative phosphorylation (Figure 5 and Figure S12). The PP_1317‐PP_1319 operon encodes a ubiquinol‐cytochrome *c* reductase and the PP_4250‐PP_4253 operon encodes a cbb3‐type cytochrome *c* oxidase. It is well known that oxidative phosphorylation has the function of oxidizing nutrients in order to produce adenosine triphosphate (ATP). That these operons are underexpressed in the presence of selenite, could reflect the metabolic failures and damage to cell function generated by the presence of this oxyanion.

During mutagenesis, we isolated other mutants with Tn5 disruptions in genes encoding proteins related to the cell membrane, namely *msbA* (a lipid transporter ABC ATPase), *wzy* (an O‐antigen polymerase) and PP_4799 (a muranoyltetrapeptide carboxypeptidase). The *msbA* and *wzy* genes form an operon in *P. putida* KT2440 and by homology with studies carried out in *P. aeruginosa* (Ghanei et al., 2007; Huszczynski et al., 2020) and other bacteria (Padayatti et al., 2019; Singh et al., 2016; Wiseman et al., 2021) it is possible that in *P. putida* both genes are related to lipopolysaccharides (LPS) trafficking and assembly. Specifically, MsbA is an essential ATP‐binding cassette transporter in Gram‐negative bacteria that flips lipid A with or without core sugars from the cytoplasmic leaflet to the periplasmic leaflet of the inner membrane (Padayatti et al., 2019). In *P. aeruginosa*, it was shown that *msbA* is an essential gene and that the activity of MsbA can be stimulated by lipid A linked to complete core oligosaccharides (Ghanei et al., 2007). The gene *wzy* encodes a polymerase responsible for the biosynthesis of polysaccharides, including LPS (Wiseman et al., 2021). Finally, a mutant in the PP_4799 gene encoding a muranoyltetrapeptide carboxypeptidase (also known as LD‐Carboxypeptidase) was obtained. Homologues in other bacterial genera indicate that these enzymes can cleave amide bonds that link an L‐amino acid to a C‐terminal D‐amino acid, and a role for these enzymes in peptidoglycan recycling has been proposed (Korza & Bochtler, 2005). Based on the results obtained in this study, it is difficult to assign a clear function to these genes (i.e. *msbA*, *wzy* and PP_4799) in the metabolism of selenium. It is likely that they participate in the transport of selenite/selenium in the cell or rather they are a response at the membrane level resulting from selenite reduction and the presence of selenium nanoparticles.

Transcriptomic experiments also revealed that exposure to selenite triggered the expression of membrane associated proteins (Figure 5). It was possible to observe in the presence of selenite the overexpression of transporters related to the uptake of amino acids (operon PP_1297, PP_1298, PP_1299 and PP_1300) and ribose (PP_2456, PP_2455). The differences in expression patterns in membrane proteins can be explained considering that selenite reduction leads to the accumulation of extracellular selenium nanoparticles, which could lead to a significant rearrangement of the membrane proteome.

## CONCLUSION

**FIGURE 8:**
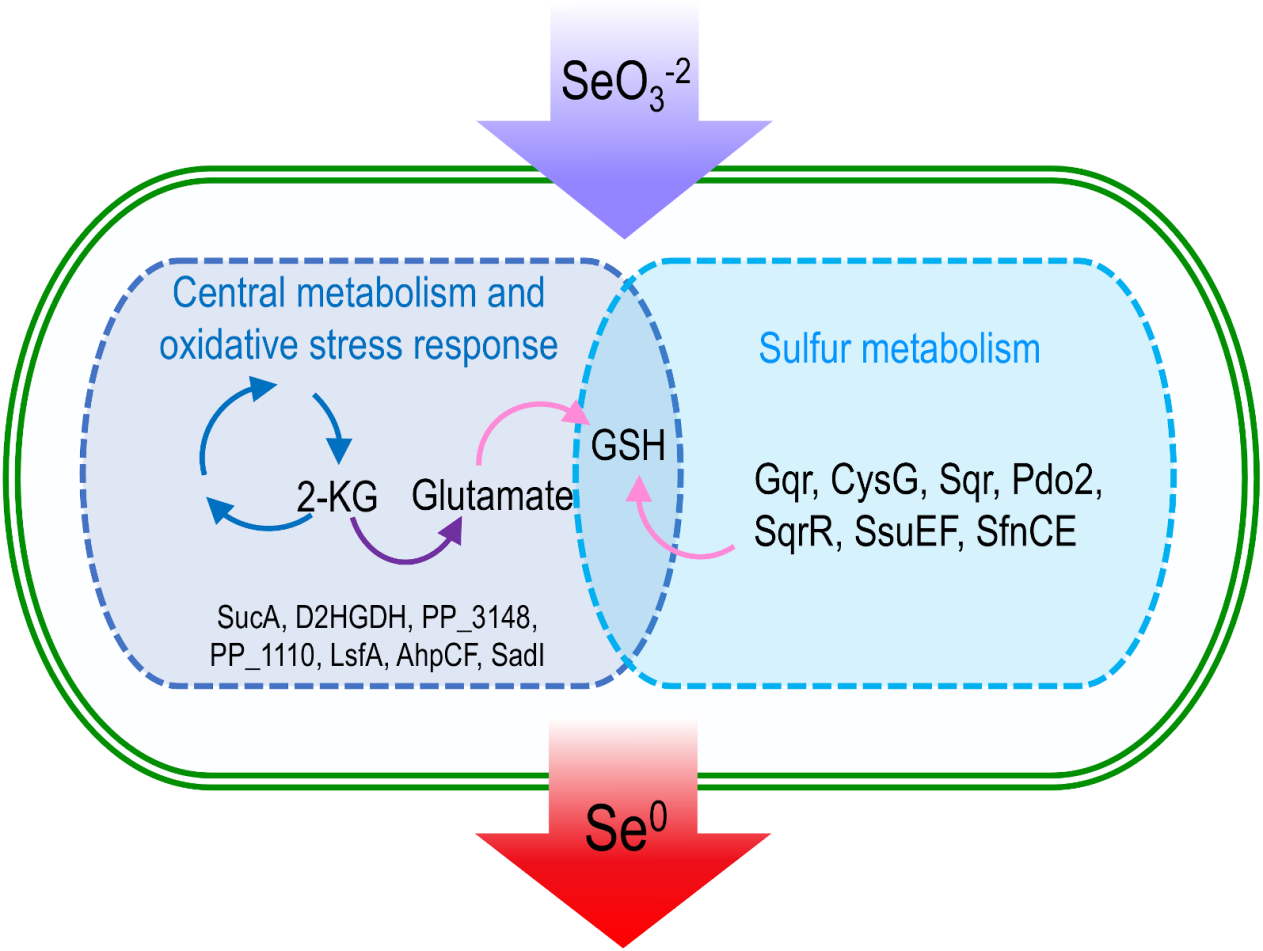
Molecular actors involved in selenite reduction in *Pseudomonas putida* KT2440. Enzymes of central metabolism, oxidative stress response and sulphur metabolism participating in the reduction of selenite to selenium nanoparticles. Both steps converge in the production of glutathione.

This work provides the first molecular insights of selenium metabolism in *P. putida* KT2440. This bacterium has a specialized central metabolism and enzymatic machinery for producing high reducing power. This characteristic makes *P. putida* an excellent bacterial model for studying chemical biotransformations that involve a reduction process, such as transforming selenite into selenium. This process is dependent on a number of coordinated cellular functions that when individually inactivated result in partial (or delayed) selenite reduction but not in its complete suppression. Our results suggest that the reduction process is carried out by an interconnected pathway involving sulphur metabolism (represented by genes *cysG*, *sqr*, *pdo2*, *sqrR*, *ssuEF and sfnCE*), 2‐oxoglutarate/glutamate metabolism (represented by genes *sucA*, D2HGDH and PP_3148) and oxidative stress response (represented by genes *Gqr*, *lsfA*, *ahpCF* and *sadI*). Genes *sucA*, D2HGDH and PP_3148 encode central metabolic enzymes related to the 2‐ketoglutarate metabolism, a key molecule and the starting point for the synthesis of glutamate and glutathione, therefore establishing a potential link between these central metabolic reactions and the response to oxidative stress. Thus, considering that sulphur metabolism is also interconnected with glutathione metabolism (Figure 7), we propose that in *P. putida* KT2440, the reduction of selenite is carried out through the metabolism of inorganic sulphur (${\mathrm{SO}}_{3}^{-2}$➔H_2_S) and the response to oxidative stress mediated by glutathione (Figure 8). This study not only sheds light on the metabolism of selenium in *P. putida* but also (i) reports the involvement of genes that had not been related to the metabolism of selenium in any other bacterial genus and (ii) highlights genes of biotechnological interest (*sqrR*, *pdo2* and *sqr*) whose suppression generates the production of selenium nanoparticles at a higher rate than the WT strain. The data provided in this study brings us closer to understanding the metabolism of selenium in bacteria, and to the development of biotechnological tools that can be used to recover this element from the environment.

## AUTHOR CONTRIBUTIONS

**Roberto Avendaño:** Formal analysis (equal); investigation (lead); visualization (equal); writing – original draft (supporting); writing – review and editing (supporting). **Said Muñoz‐Montero:** Formal analysis (equal); visualization (equal); writing – original draft (supporting); writing – review and editing (supporting). **Diego Rojas‐Gätjens:** Formal analysis (equal); investigation (supporting); visualization (equal); writing – original draft (supporting); writing – review and editing (supporting). **Paola Fuentes‐Schweizer:** Formal analysis (supporting); investigation (supporting); writing – original draft (supporting); writing – review and editing (supporting). **Sofia Vieto:** Formal analysis (supporting); investigation (supporting); writing – original draft (supporting); writing – review and editing (supporting). **Rafael Montenegro:** Formal analysis (supporting); investigation (supporting); writing – original draft (supporting); writing – review and editing (supporting). **Manuel Salvador:** Formal analysis (supporting); investigation (supporting); writing – original draft (supporting); writing – review and editing (supporting). **Rufus Frew:** Formal analysis (supporting); investigation (supporting); writing – original draft (supporting); writing – review and editing (supporting). **Juhyun Kim:** Formal analysis (supporting); investigation (supporting); writing – original draft (supporting); writing – review and editing (supporting). **Max Chavarría:** Conceptualization (equal); formal analysis (equal); funding acquisition (equal); project administration (equal); supervision (equal); visualization (equal); writing – original draft (equal); writing – review and editing (equal). **Jose I. Jiménez:** Conceptualization (equal); formal analysis (equal); funding acquisition (equal); project administration (equal); supervision (equal); visualization (equal); writing – original draft (equal); writing – review and editing (equal).

## CONFLICT OF INTEREST

The authors declare no competing financial interests.

## Supporting information


Appendix S1.

Figure S1.

Figure S2.

Figure S3.

Figure S4.

Figure S5.

Figure S6.

Figure S7.

Figure S8.

Figure S9.

Figure S10.

Figure S11.

Figure S12.

Figure S13.

Figure S14.

Figure S15.

Video S1.

Data S1.

Data S2.
