# Supplementary figures and images for: Production of selenium nanoparticles occurs through an interconnected pathway of sulphur metabolism and oxidative stress response in Pseudomonas putida KT2440
Source: Microb Biotechnol. 2023 Jan 22;16(5):931–46. doi: 10.1111/1751-7915.14215 (PMC10128140; doi:10.1111/1751-7915.14215)

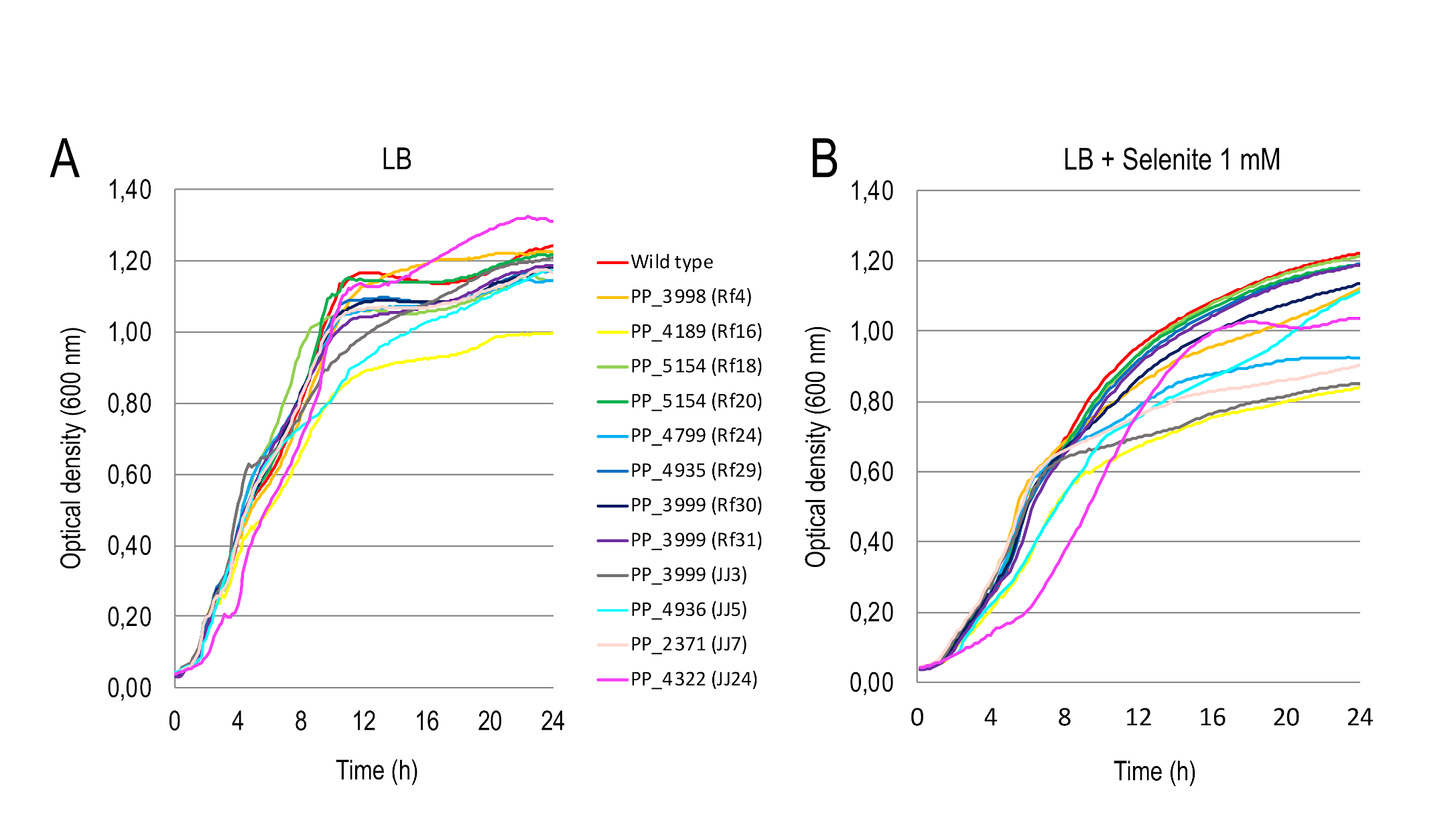

Supplement: Supplementary file 2 — Figure S1. [file MBT2-16-931-s012.tif]

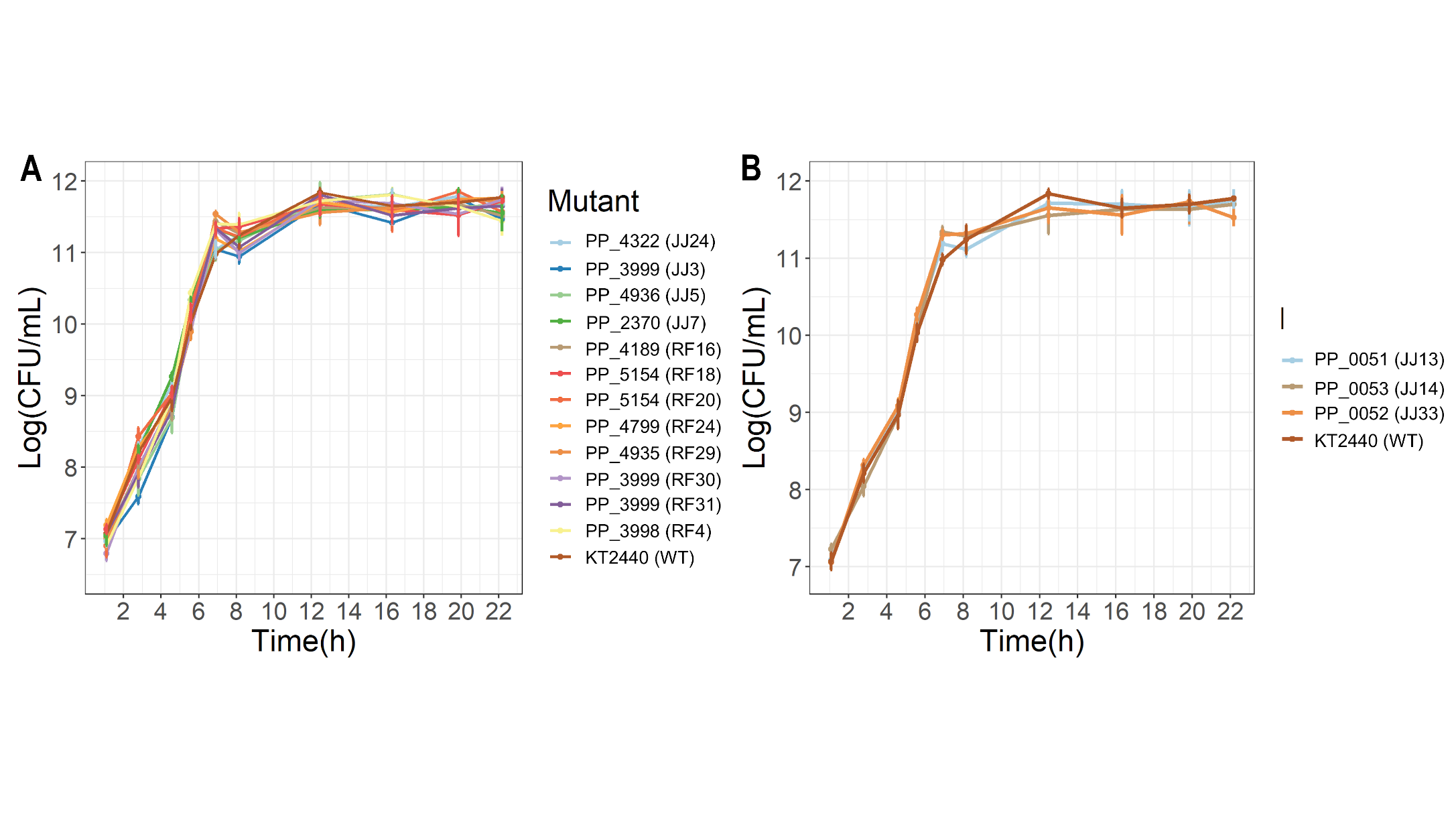

Supplement: Supplementary file 3 — Figure S2. [file MBT2-16-931-s017.tif]

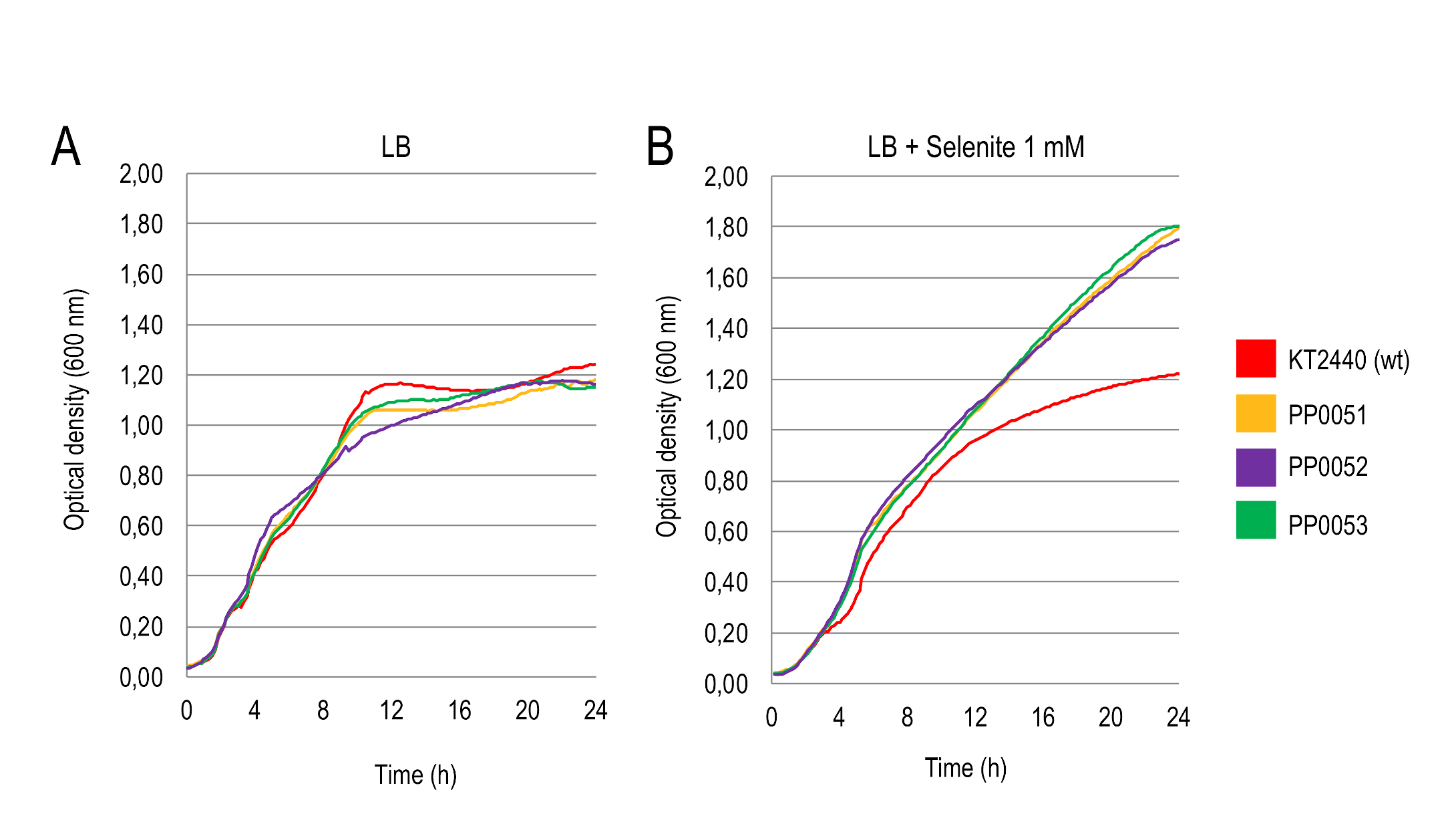

Supplement: Supplementary file 4 — Figure S3. [file MBT2-16-931-s007.tif]

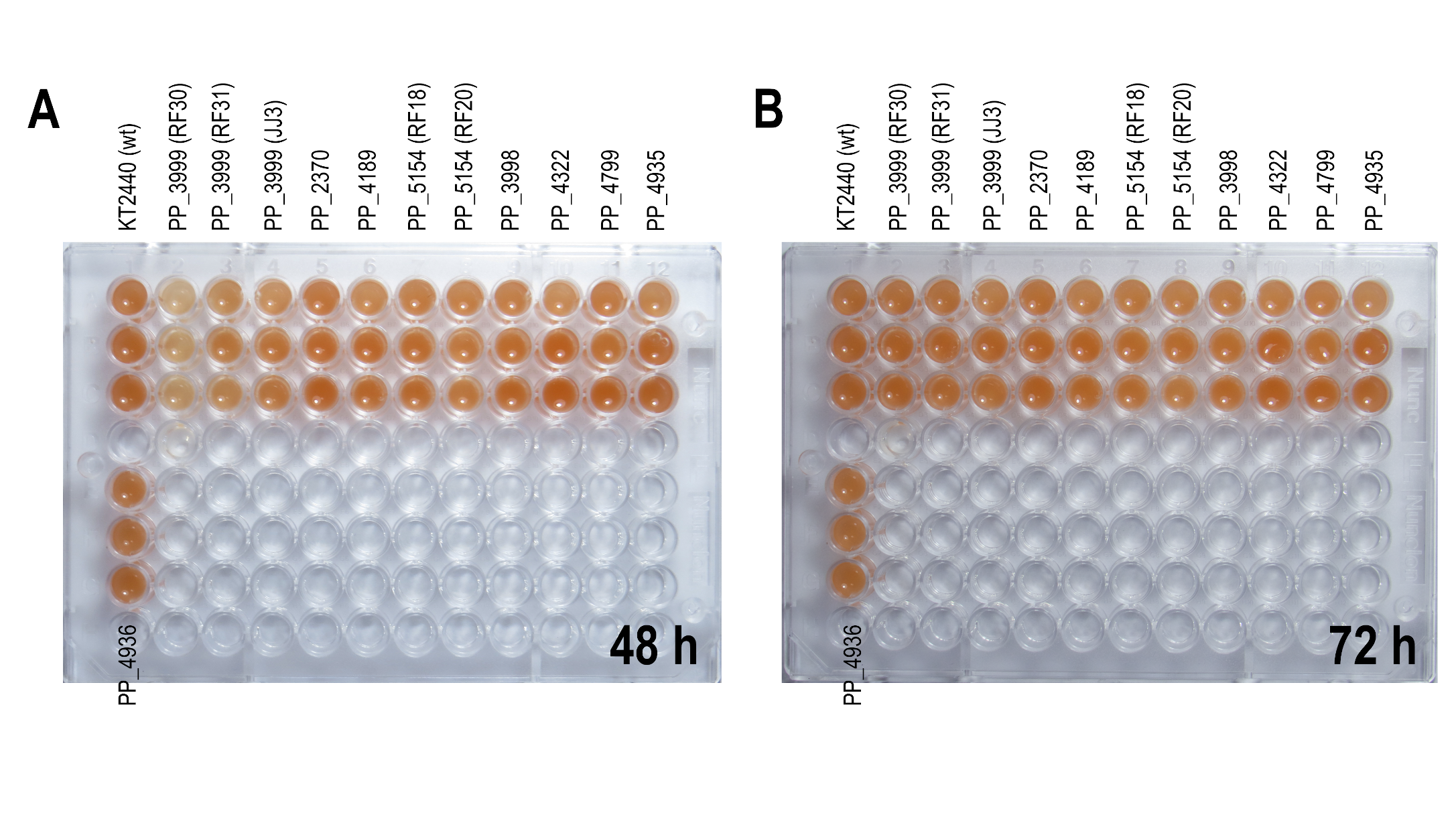

Supplement: Supplementary file 5 — Figure S4. [file MBT2-16-931-s018.tif]

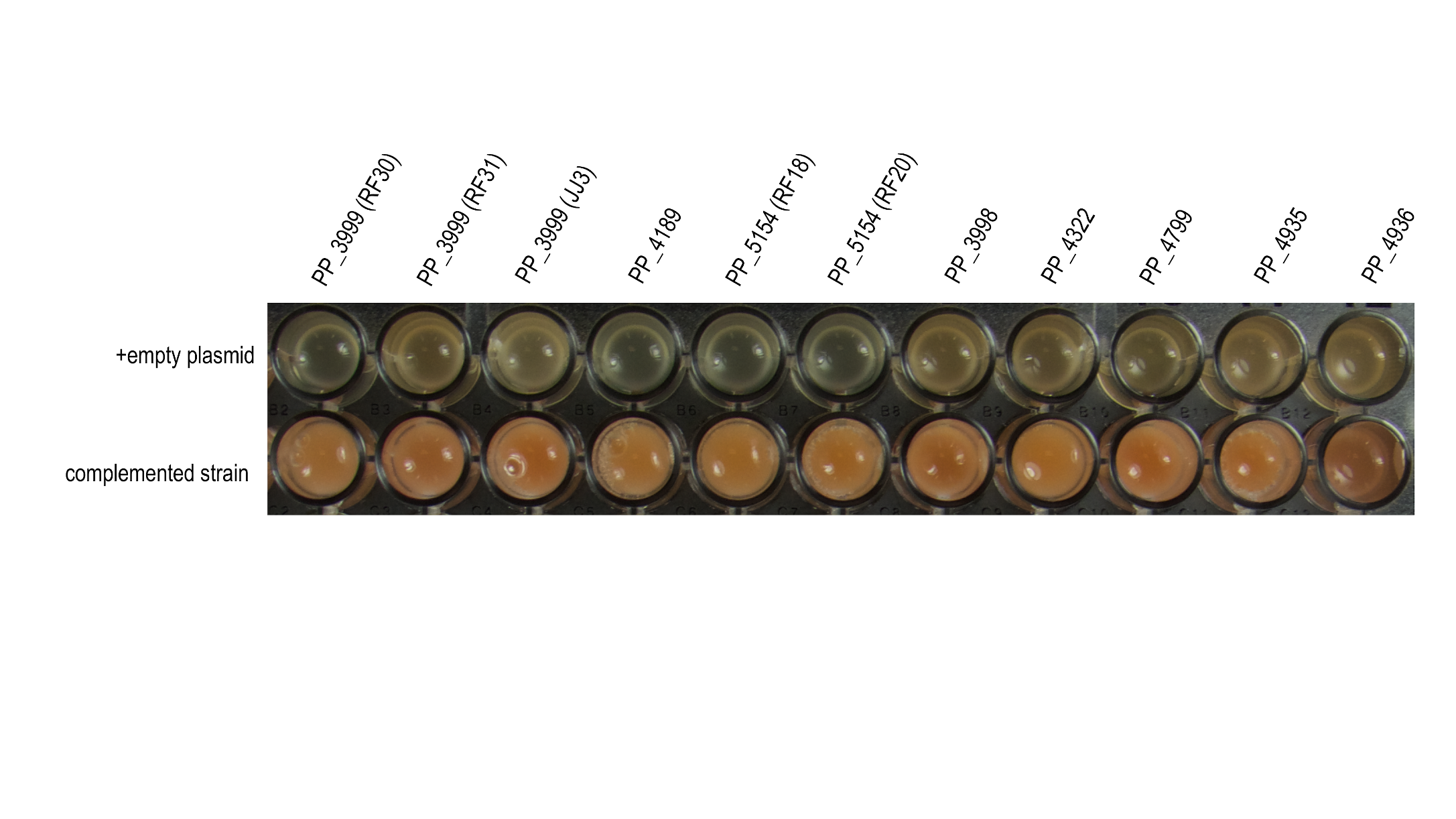

Supplement: Supplementary file 6 — Figure S5. [file MBT2-16-931-s015.tif]

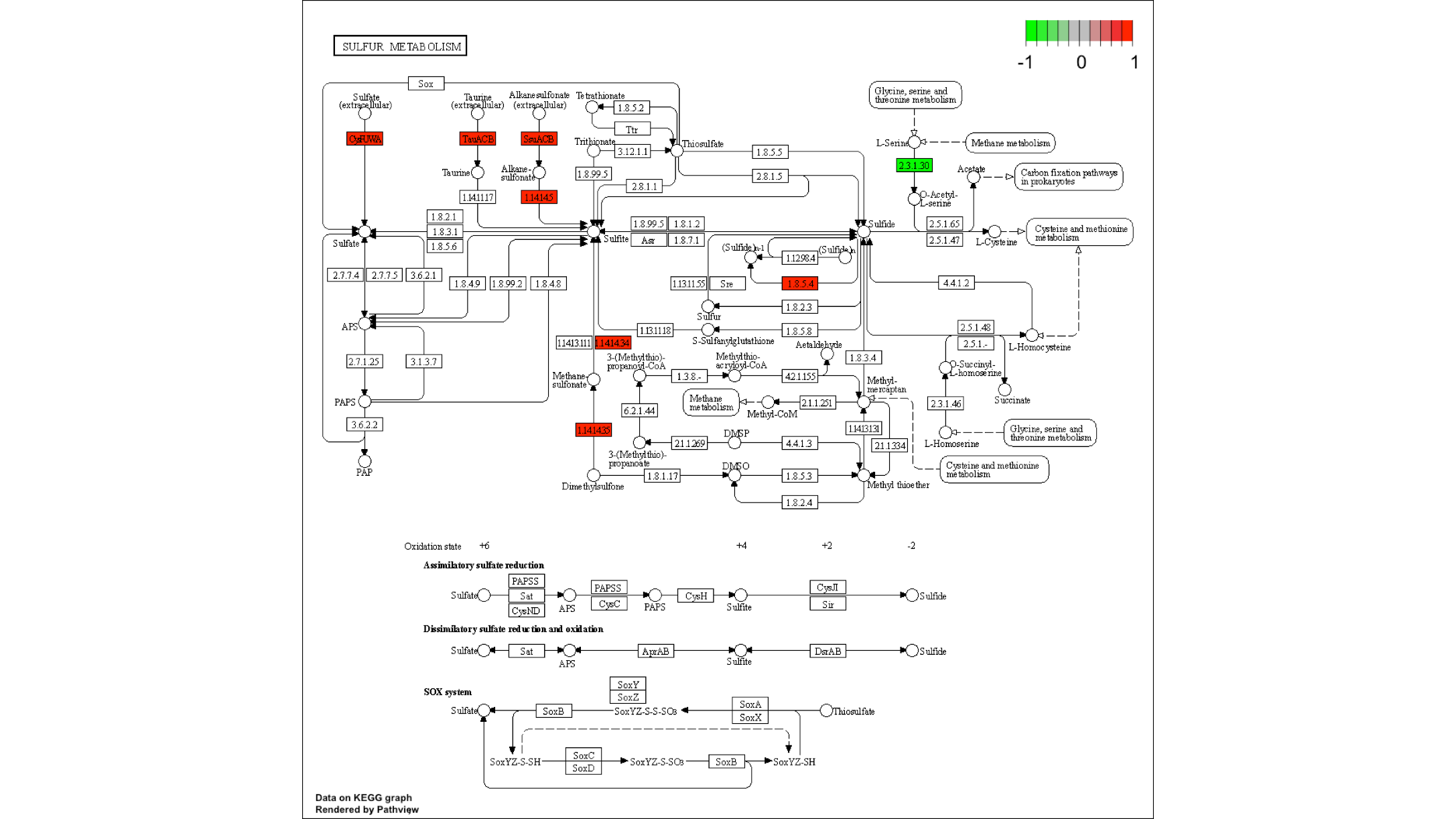

Supplement: Supplementary file 7 — Figure S6. [file MBT2-16-931-s011.tif]

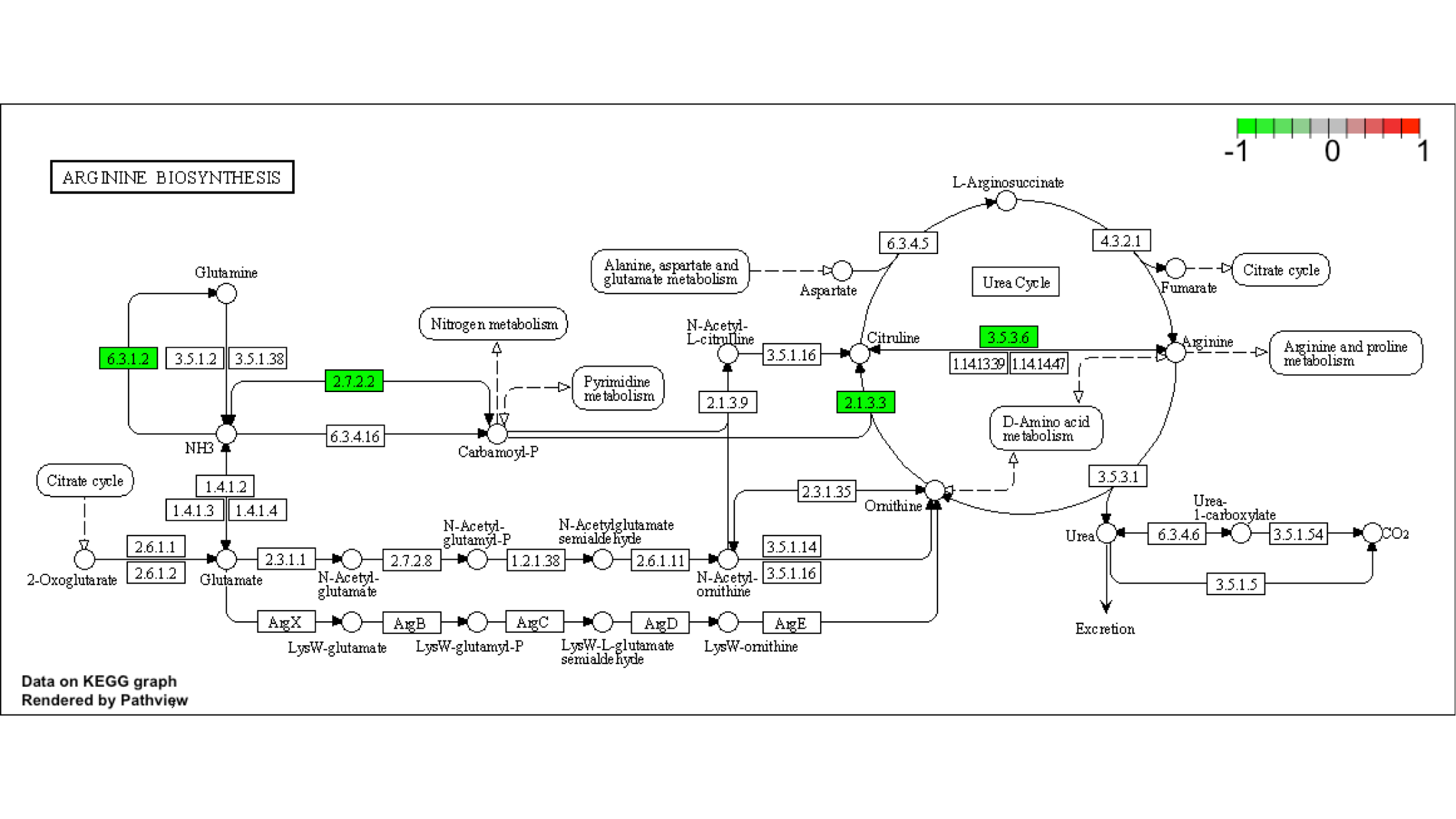

Supplement: Supplementary file 8 — Figure S7. [file MBT2-16-931-s010.tif]

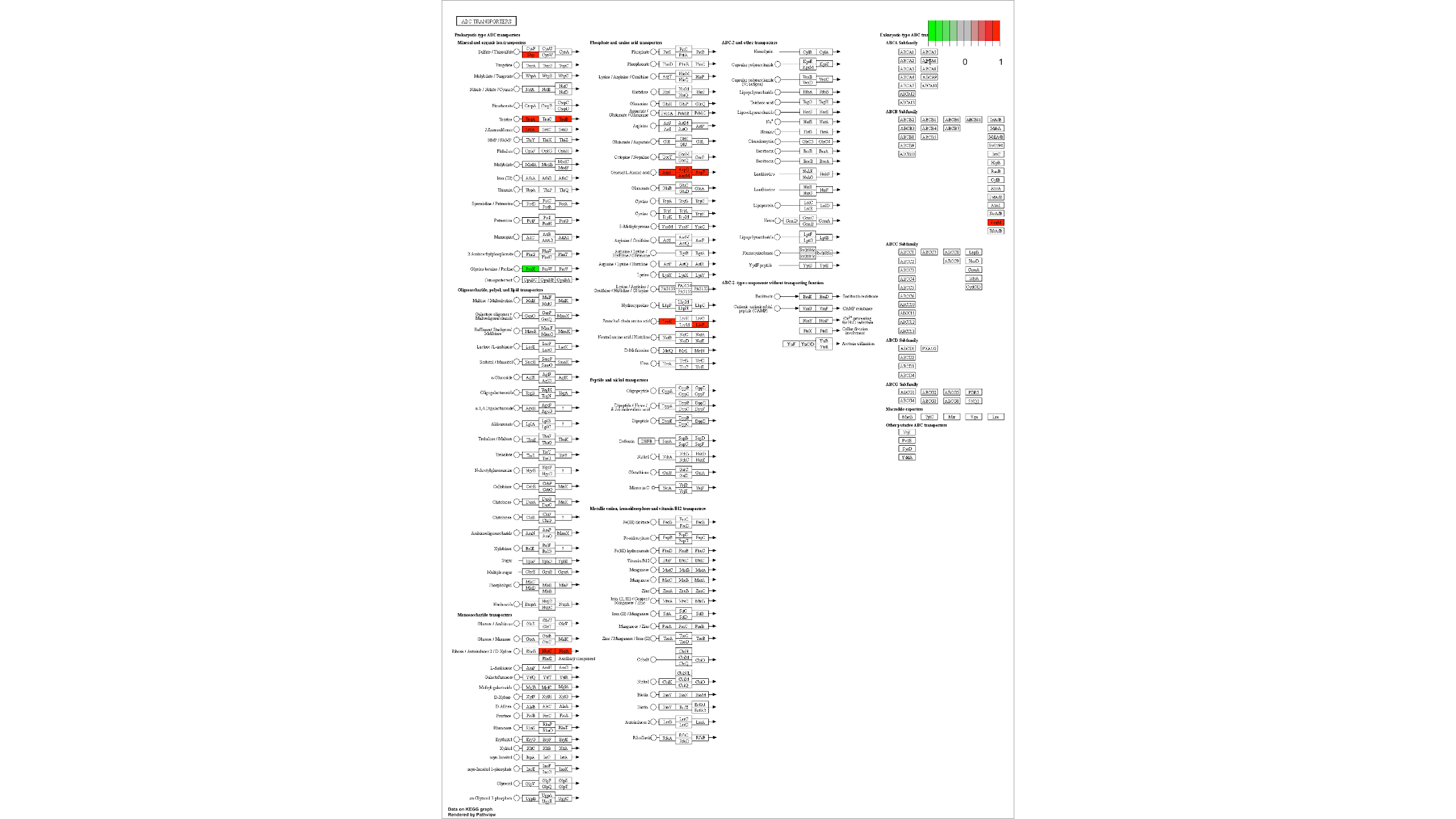

Supplement: Supplementary file 9 — Figure S8. [file MBT2-16-931-s003.tif]

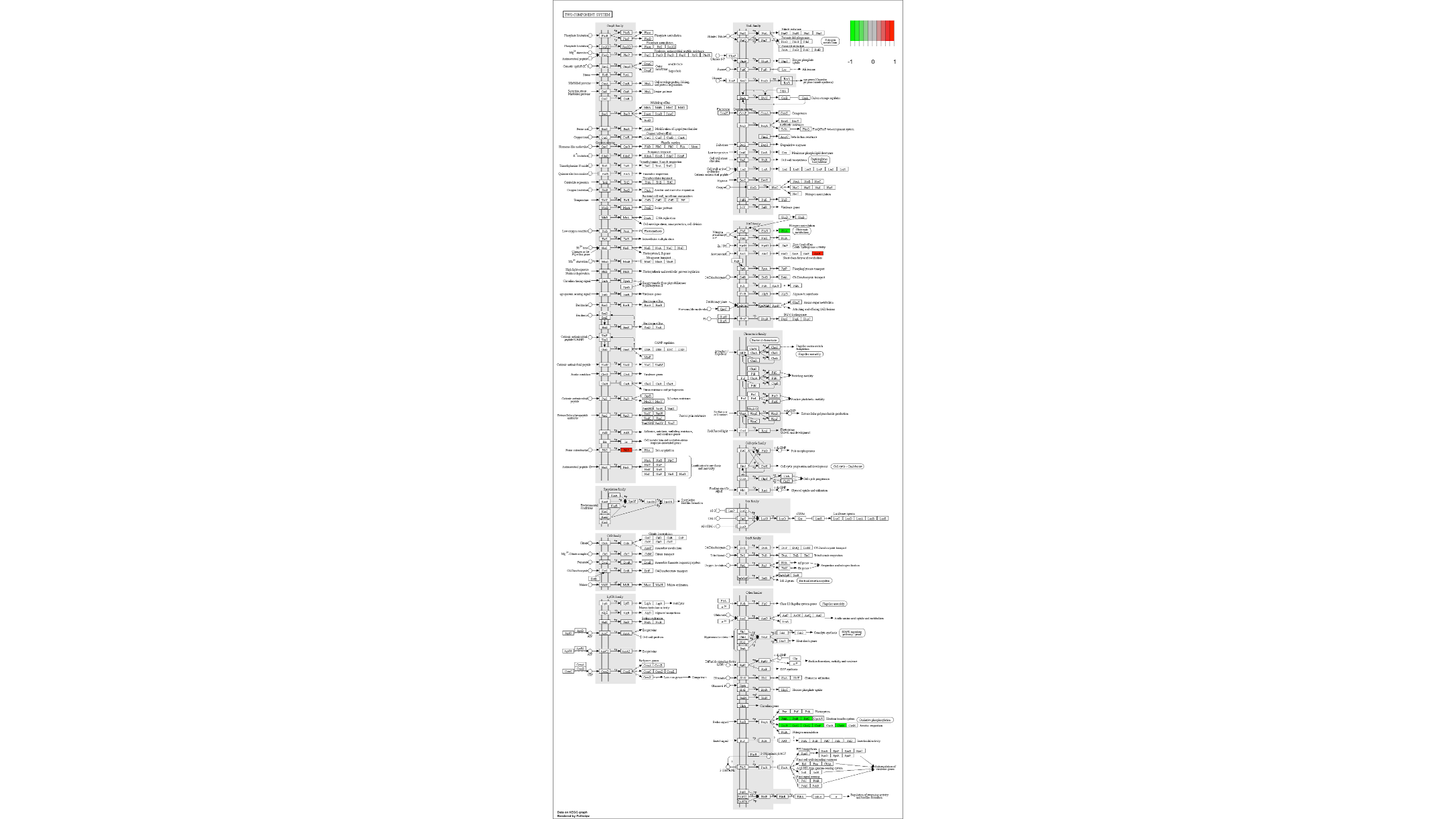

Supplement: Supplementary file 10 — Figure S9. [file MBT2-16-931-s019.tif]

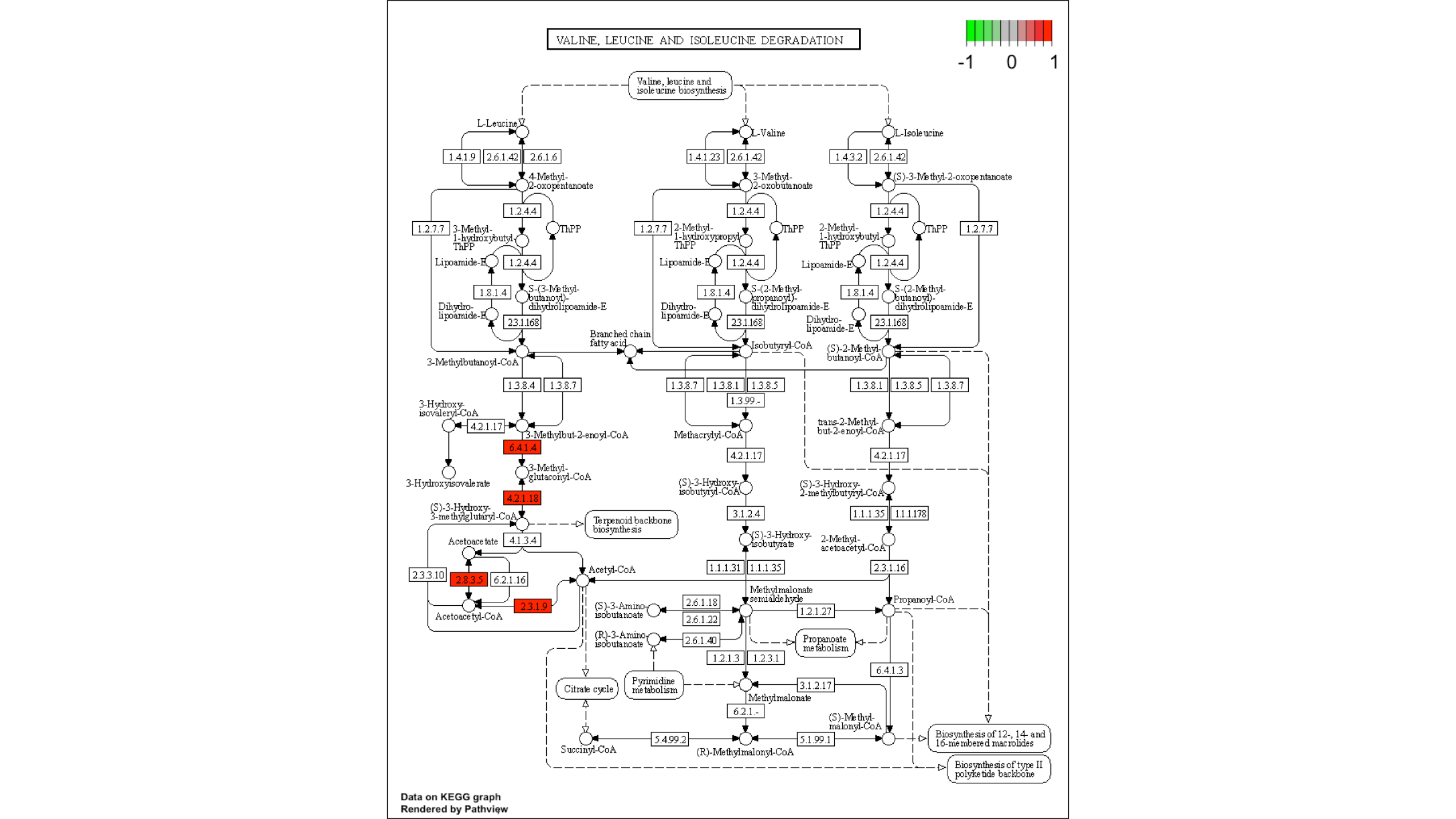

Supplement: Supplementary file 11 — Figure S10. [file MBT2-16-931-s016.tif]

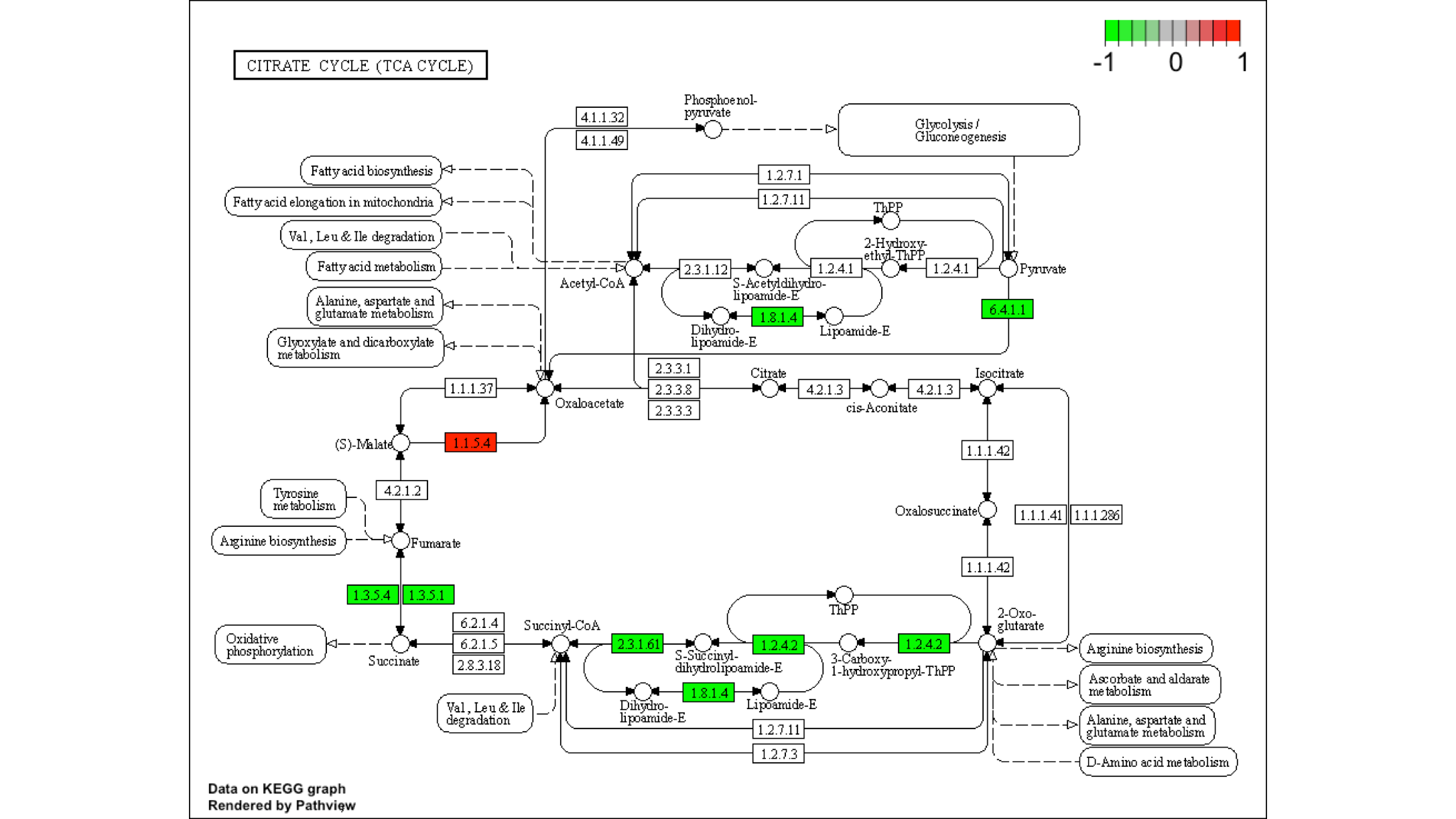

Supplement: Supplementary file 12 — Figure S11. [file MBT2-16-931-s014.tif]

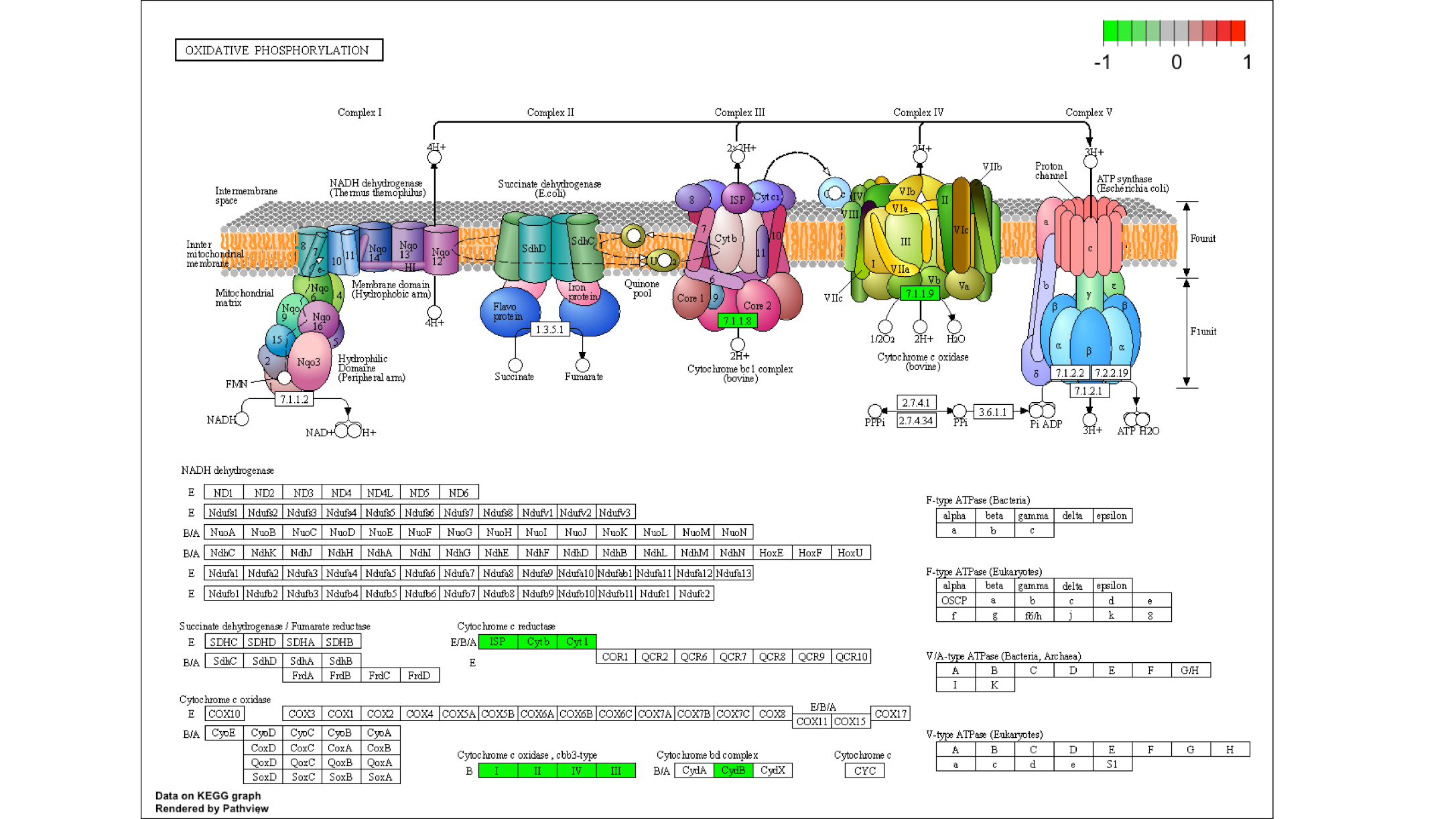

Supplement: Supplementary file 13 — Figure S12. [file MBT2-16-931-s013.tif]

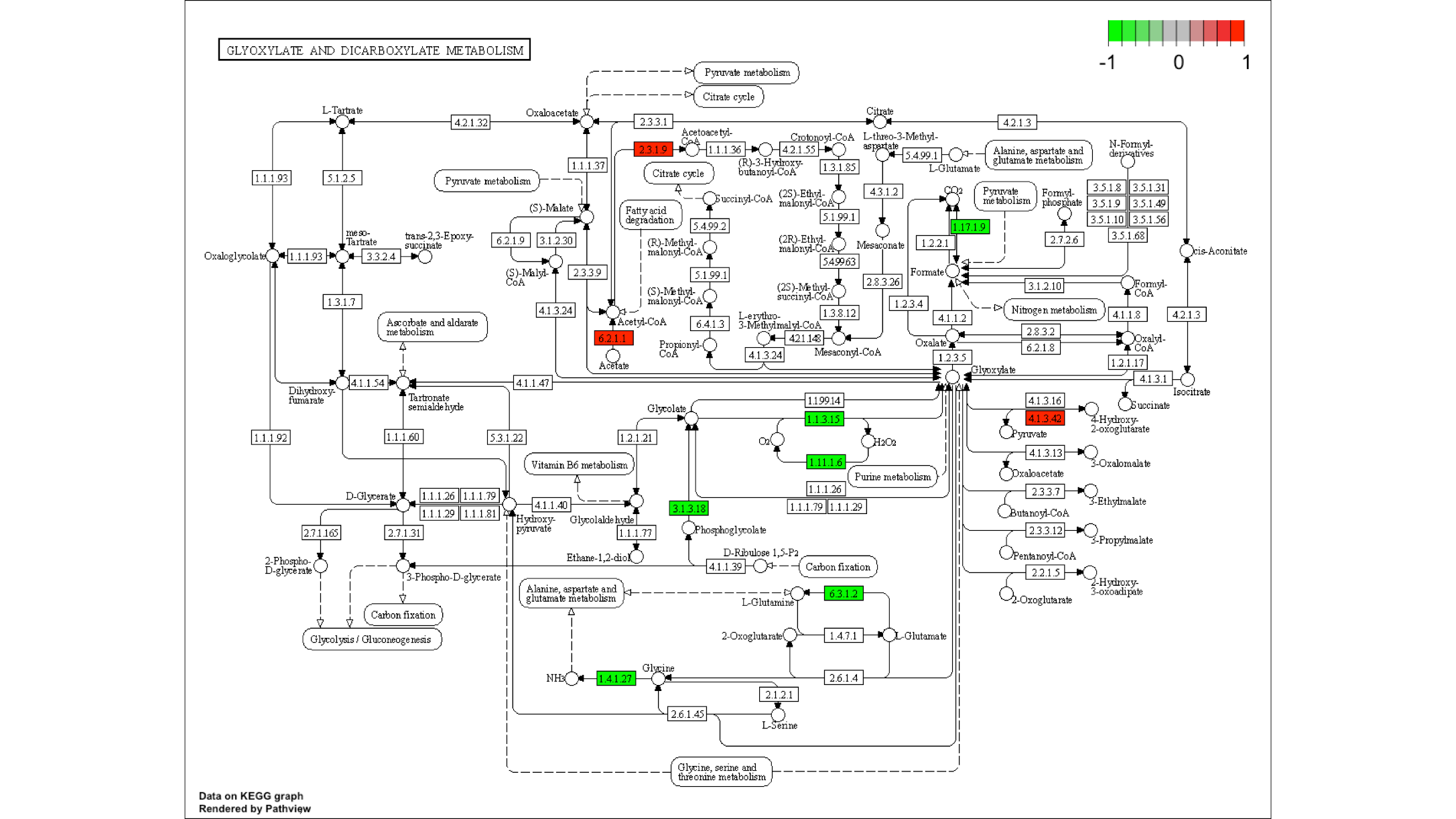

Supplement: Supplementary file 14 — Figure S13. [file MBT2-16-931-s001.tif]

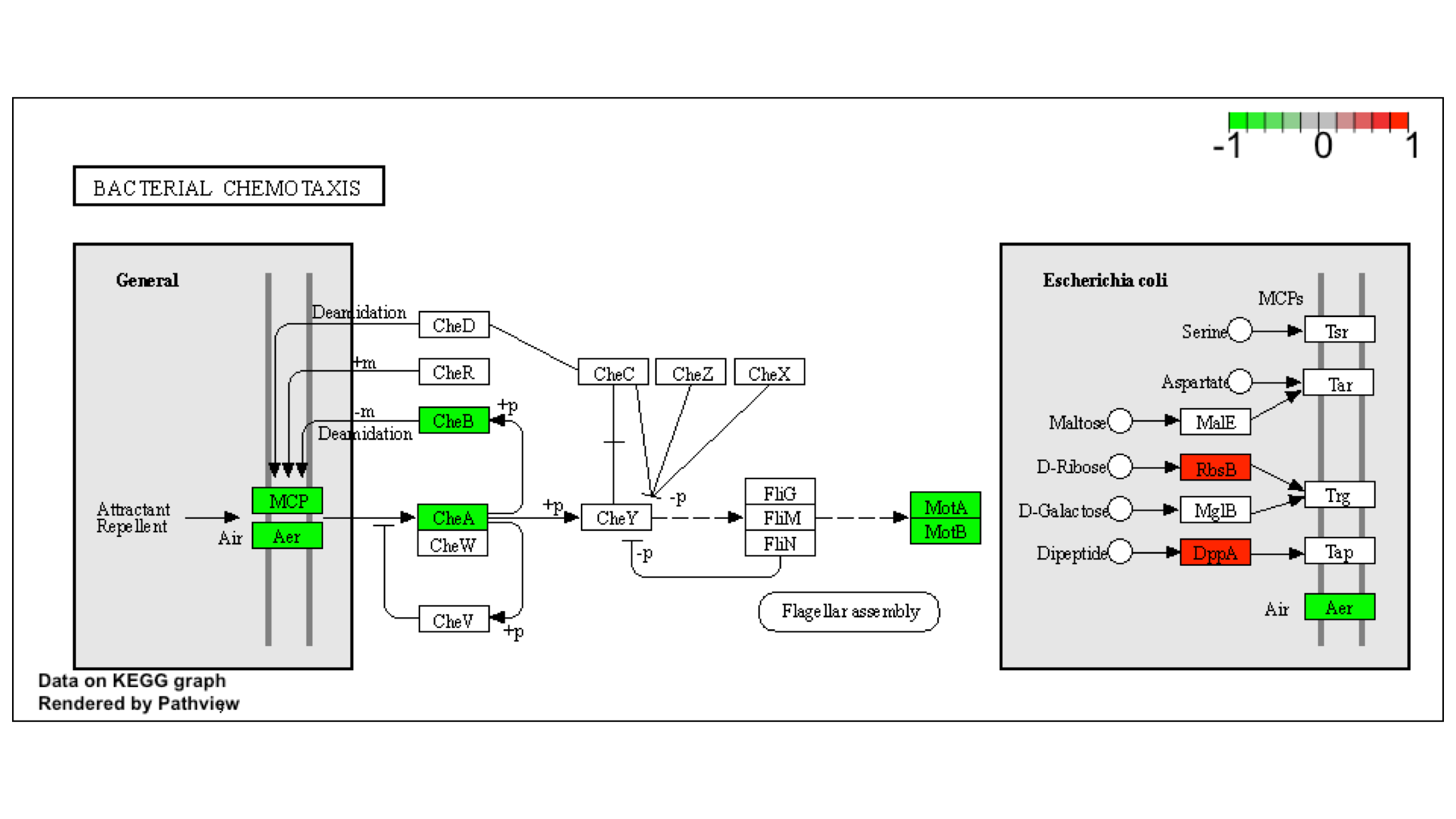

Supplement: Supplementary file 15 — Figure S14. [file MBT2-16-931-s009.tif]

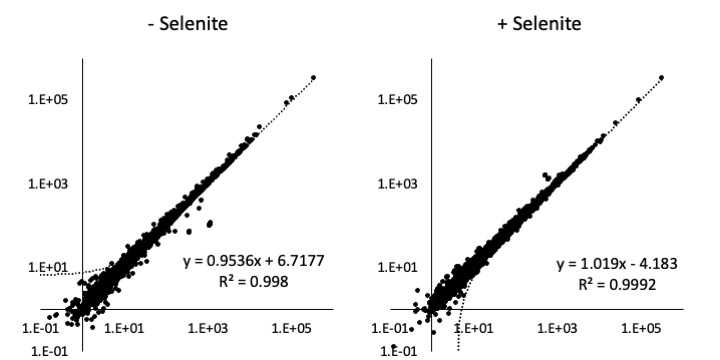

Supplement: Supplementary file 16 — Figure S15. [file MBT2-16-931-s002.tiff]
